# Supplementary material for: Far away or yesterday? Shifting perceptions of time for political ends
Source: PLoS One. 2022 Nov 3;17(11):e0277179. doi: 10.1371/journal.pone.0277179 (PMC9632895; doi:10.1371/journal.pone.0277179)

**Far away or yesterday? Shifting perceptions of time for political ends**

**SUPPORTING INFORMATION**

Andrew Dawson, Scott A. Leith, Cindy L.P. Ward, Sarah Williams and Anne E. Wilson

Wilfrid Laurier University

# **Shifting perceptions of time for political ends - Supporting Information**

The current document reports the three precursor studies, then lists any additional information for each of the main studies that were included in the main paper. Tables and figures specific to this document are included at the end.

## **Use of Prior Data**

Precursor Studies 1-3 make use of data from a prior set of studies investigating implicit theories of change [14]. In these studies, item(s) measuring subjective temporal distance were included after the primary study materials, on a separate page (either paper or web). Subjective temporal distance was measured as an exploratory variable in these studies. The subjective time measure came after the central dependent measures (implicit theories of change), was theoretically irrelevant to the research question of the first paper (which already contained 7 studies on a wider range of topics than politics), and did not modify any of the conclusions drawn in Leith et al [14]. Beyond the descriptive statistics of the samples, all analyses reported in the supporting information are novel and were not included in Leith et al. [14]. The decision to include Precusor Studies 1-3 was not made lightly. Although it is not uncommon for data pertaining to more than one distinct research question to be collected in a single study and reported separately, we are cautious of the gratuitous practice of carving up a data set (“salami slicing”), especially when it is not transparent. Because of these concerns, we considered simply excluding the earlier studies that are partially reported in Leith et al. [14]. However, these studies do offer tests of the same hypothesis described in the main paper, and for different points in time. It would have been impossible to return to the specific moments in time that the data was collected (in particular, the 2011 and 2014 Canadian federal election and, less precisely, a point in Obama’s presidency). A strength of this manuscript is that it examines real-world political contexts over the course of several leaders and elections. Sampling a variety of different political contexts over these additional years increases generalizability that would be lost were we to omit Studies 1-3. As a result, we determined that inclusion of all studies, along with clear disclosure of the past manuscript reporting aspects of Studies 1-3 *other than subjective time*, would be the most open, informative, and ethical solution. We are of course receptive to feedback or editorial direction about this decision and how we address it.

## **Exploratory Analyses**

Much of the additional information included in this supplement describes exploratory analyses that are not meant to be the focus of the paper and were not central to our hypotheses. Much of these analyses are correlational, and path models test only one possible theorized causal direction. We emphasize of course that causality cannot be determined from any such analyses and additional research would be required to test each step of these models. They are included, with appropriate caveats, for additional information and because they could be generative of future research directions. Overall the results in this section should be interpreted with caution.

# **Precursor Study 1**

The first study was conducted within the final two weeks leading up to the 2011 Canadian Federal Election, in which Conservative party (right-wing) leader and incumbent Stephen Harper faced off against Liberal leader (center-left) Michael Ignatieff. During this election, attack ads included a number of unflattering, often chronologically distant in time, statements made by candidates. We predicted that participants would relegate damning quotations made by a favored candidate to the more distant past to diminish their relevance to the present. Conversely, we expected that participants might actively pull the unflattering quotations made by an opposing candidate to be subjectively nearer in time, allowing them to use past foibles to cast aspersions on a candidate’s current standing.

**Method**

***Participants and Power***

Two hundred and twenty-six participants were recruited from local farmers' markets in Southern Ontario, Canada, within the two weeks before the last Canadian federal election. Participants were offered a large candy bar as compensation. Ethics approval was obtained from the Wilfrid Laurier University Research Ethics Board (Reference #3953). Participants provided written consent.

It is important to note that the Canadian political system includes multiple parties, but that historically, only two parties have succeeded as the governing party: the Liberal and Conservative parties. Because we were examining only the two largest of Canada’s four prominent federal parties (Liberals and Conservatives), we determined *a priori* to exclude participants who supported one of the two smallest parties (NDP and Green), who would not review their candidate. This allowed us to define all participants as either a *supporter* or *opponent* of the candidate they reviewed (third parties cannot be as readily classified in this manner). Participants were also excluded for leaving the main questions blank. In the end, 126 participants were used in the final analyses (64 Liberal, 62 Conservative; 66 female, 57 male, 3 undisclosed; *M*_age_ = 45.61, SD = 15.75). According to G*Power 3.1 [15] this sample size was sufficiently sensitive to detect an effect size of *f* = 0.25 (*η_p_²* = 0.06) with 80% power and α = 0.05.

***Procedure***

Participants were recruited for a study on voting in Canada and completed the paper-and-pencil survey privately near the researchers’ booth. Participants reported which party they would vote for if the election was to be held at that moment.

Participants were then assigned to one of two conditions: the Stephen Harper condition (the Conservative leader) or the Michael Ignatieff condition (the Liberal leader), and asked to read five personally unflattering quotations made by that party leader in the relatively distant past (all quotes were uttered between 5-15 years earlier; with an average of 10 years for both candidates; see Table S1 for quotes and description).

Participants were randomly assigned to read either past unflattering quotations from one of the two candidates: the Liberal party leader, Michael Ignatieff, or the leader of the Conservative party, Stephen Harper; participants were told the quotes were from ‘an average of 10 years ago.’ (Individual quote dates were not provided to the participants). Because we used actual statements uttered by both candidates, they by necessity were qualitatively different, but they reflected the predominant criticisms levied at each candidate (see Appendix). For example, the Liberal leader was criticized for being an outsider and uncommitted to Canada, while the Conservative leader was criticized for being unempathetic and unsupportive of Canadian social safety nets.

After reading the five assigned quotations, participants were asked to rate the overall valence of the quotations from -3 (*very negative*) to 0 (*neither positive or negative*) to +3 *(very positive*). Following this, participants responded to a candidate-specific version of the standard general personality implicit theories scale (as described in Leith et al., [14]). Next, participants were asked to report their level of agreement with two statements designed to capture the degree to which participants felt that the candidate’s past actions should reflect the candidate’s standing in the present. The two items were combined to provide an overall measure of current relevance (*r_SB_* = .79). On the back page of the survey was an item to assess subjective perceptions of time: “Sometimes events feel far or close in time, regardless of when they actually occurred. How far away in time do [the leader’s] statements feel to you?” (*1 = Very close; 10 = Very distant;* [1]). Finally, participants filled out a demographics measure.

**Results and Discussion**

***Subjective Temporal Distance***

A 2 (candidate: Liberal vs. Conservative) × 2 (voter affiliation: Liberal vs. Conservative) ANOVA examining subjective temporal distance revealed a significant interaction, *F*(1, 122) = 38.90, *p* < .001, *η_p_^2^* = .24. Within the Conservative leader condition, Conservative voters rated their leader’s damning quotes as significantly further away in time than Liberal voters, *F*(1,122) = 13.56, *p* < .001, *η_p_^2^* = .10. Within the Liberal leader condition, Conservative voters rated the Liberal leader’s damning quotes as significantly closer in time than Liberal voters, *F*(1,122) = 27.08, *p <* .001, *η_p_^2^* = .18. As predicted, both Liberals, *F*(1,122) = 31.89, *p* < .001, *η_p_^2^* = .21, and Conservatives, *F*(1,122) = 10.06, *p* = .002, *η_p_^2^* = .08, pulled the negative quotes of the opposing candidate closer, while pushing their favored candidate’s quotes away. Estimated marginal means and standard errors for this analysis can be found in Table S2.

***Path Analysis***

We expected that participants were manipulating the subjective temporal distance of political events to increase or decrease their relevance to the current election – enhancing or attenuating the harm the negative quotations could deal to a candidate’s standing. We did not predict full mediation; political views are complex and include many variables beyond how far in the past something feels.

To test this process, we conducted a moderated mediation using Model 8 of the SPSS macro PROCESS [17], conducted with 5,000 bootstrap samples. Candidate affiliation (Liberal vs Conservative) was entered as our predictor variable, candidate affiliation (Liberal vs Conservative) as the moderator, subjective temporal distance as the mediator, and perceived relevance as the outcome. The full model is presented in Figure S1. Standardized z-scores were used for all continuous variables, a practice continued for all path analyses throughout this document as well as the main paper. We found that the interaction between voter affiliation and candidate affiliation did indeed predict subjective time (a path, ß = -1.92, *t*(122) = -6.24, *p* < .001), which in turn predicted present relevance (b path, ß = -0.52, *t*(121) = -6.92, *p* < .001). Bias-corrected bootstrapping indicated that our index of moderated mediation was significant (index = 1.00, 95% CI = 0.57, 1.50). We also examined the conditional indirect effects. For Liberals, the quotes seemed farther away when they were spoken by Ignatieff, which in turn made them seem less relevant (effect = -0.64, 95% CI = -0.99, -0.34). For conservatives, the quotes seemed farther way when they were spoken by Harper, which in turn made them seem less relevant (effect = 0.36, 95% CI = 0.11, 0.64).

# **Precursor Study 2**

Precursor Study 1 focused solely on the past failures of two candidates, as perceived by opponents and supporters. Precursor Study 2 expands on this finding by testing voter reactions to a candidate’s past success as well as failure. We predicted that voters would perceive a past failing of a favored candidate to be in the distant past, whereas an equidistant success would be perceived as more recent in time. Opponents of the candidate should display the opposite pattern. To increase the generalizability of our findings, we turned to a different political context: this study was conducted at the end of 2011 and focused on (then) U.S. President Barack Obama of the center-left Democratic Party. At that time of the study there was no clear Republican candidate, thus all participants evaluated Obama. We a priori defined Democrat voters as favoring Obama, Republican voters as opposing him, and third-party/independent voters as to be excluded from the sample since they could not be clearly classified in this manner.

Additionally, in Precursor Study 2 we sought to increase experimental control by holding the stimuli constant. Rather than using real world acts committed by the political leader (which are ecologically valid but qualitatively different across conditions), we fabricated two evaluations of President Obama’s performance as Senator (one positive and one negative), described as bi-partisan and approved with a high degree of agreement between Democrat and Republican evaluators. In this way, the content of the positive and negative stimuli were matched.

**Method**

***Participants and Power***

Two-hundred and fifty participants recruited from Amazon’s Mechanical Turk participated in our online study in exchange for $0.40. Ethics approval was obtained from the Wilfrid Laurier University Research Ethics Board (Reference #2788). Participants provided written consent. Participants were excluded for failing an attention check. Because this study examined the effects of party loyalty and political bias, the independent and undisclosed participants, that is those who did not identify with either the Republican or Democrat parties, were also removed. Finally, those who were not highly affiliated with their chosen party, that is those who gave the lowest possible score on a measure of affiliation (1), were excluded as well. The final sample contained 187 U.S. citizens (120 Democrat, 67 Republican; 109 female, 78 male; *M*_age_ = 37.75, *SD* = 14.22, Range 18-73). According to G*Power 3.1 [15] this sample size was sufficiently sensitive to detect an effect size of *f* = 0.20 (*η_p_²* = 0.04) with 80% power and α = 0.05.

***Procedure***

This study was completed on-line in one 15-minute session. Participants first completed demographics information, which included questions regarding age, gender and political orientation (i.e., “If a federal election were held tomorrow, which political party would you vote for?”). Participants also indicated the strength of their association to the political party on a Likert-type scale anchored at 1 *(not strongly associated)* to 7 *(very strongly associated)*.

Participants were randomly assigned to read a negative or positive representation of Barack Obama’s time as Senator (2005 - 2008). Both representations were framed as bi-partisan reviews, jointly conducted by Republicans and Democrats with ‘strikingly high bi-partisan consensus.’. It was indicated that all Senators during the same time-frame had been evaluated. In the positive condition participants read that the committee concluded that ‘Barack Obama was often successful in his efforts’, and that ‘his behavior was consistent with his core goals: the support of working families and the betterment of America.’; Barack Obama’s grade was an ‘A’. In the negative condition, participants read that the committee concluded that ‘Barack Obama was often ineffectual in his efforts,’ and that, ‘his behavior often fell far short of his core goals. Rather, his actions often led to an increase in the unfair powers of big industry and the mega-rich;’ Barack Obama’s grade was a ‘C -’.

At the outset, participants were asked to rate their overall perceived valence of the evaluation, on a scale from -3 (*very negative*) to 0 (*neither positive or negative*) to +3 *(very positive*). Participants then responded to a modified version of the general person implicit theory scale (see Leith et al., [14]), and the item “How much do you think Obama’s track record as Senator reflects his *current views*?” (*1 = Not At All Representative; 7 = Very Much Representative*). Finally, on a separate web page, participants completed a measure of subjective temporal distance, in which they moved an arrow along a slider bar to indicate how far away Barack Obama’s time as Senator *felt*; the endpoints were “Feels very close” (0) and “feels very distant” (100).

**Results and Discussion**

***Subjective Temporal Distance***

A 2 (condition) × 2 (participant party) ANOVA examined the impact on subjective temporal distance. There was a significant interaction between the two factors, *F*(1, 179) = 22.38, *p* < .001, *η_p_^2^* = .11. For Democrats, a negative evaluation of Obama’s senate tenure made it feel significantly farther away than when it was positively evaluated, *F*(1, 179) = 4.69, *p* = .032, *η_p_^2^* = .03. Republicans showed the opposite pattern, where a negative evaluation of Obama’s senate tenure made it feel significantly *closer* in time than when it was positively evaluated, *F*(1, 179) = 18.42, *p* < .001, *η_p_^2^* = .09. As predicted, Democrats protected Obama by showing the reverse tendency to distance his failings and keep close his successes, while Republicans used subjective time to attack his record by seeing (equidistant) failures as relatively recent. Estimated marginal means and standard errors for this analysis can be found in Table S3.

***Path Analysis***

We expected that the interaction between candidate and party allegiance would predict the subjective temporal distance of Obama’s Senatorial performance, which in turn would affect how much participants believed his past performance reflected the President Obama of the present. To test this process, we conducted a moderated mediation analysis using the same PROCESS design as in Precursor Study 1. Valence condition was entered as our predictor variable, voter affiliation (Democrat vs Republican) as the moderator, subjective temporal distance as the mediator, and perceived relevance as the outcome. The full model is presented in Figure S2. We found that the interaction between voter affiliation and valence did indeed predict subjective time (a path, ß = -1.40, *t*(177) = -4.83, *p* < .001), which in turn predicted present relevance (b path, ß = -0.18, *t*(176) = -2.56, *p* = .011). Bias-corrected bootstrapping with 5,000 samples indicated that our index of moderated mediation was significant (index = 0.25, 95% CI = 0.05, 0.51). For Democrats, a negative evaluation felt farther way, and in turn felt less relevant (effect = -0.71, 95% CI = -1.04, -0.38), while for Republicans, a *positive* evaluation felt farther away, and therefore less relevant (effect = 0.18, 95% CI = 0.04, 0.37).

# **Precursor Study 3**

Precursor Study 3 again takes place in Canada, in a new electoral cycle: the lead-up to the 2015 Federal Election. Stephen Harper was again the (Conservative) incumbent; the new Liberal candidate was Justin Trudeau. This study was conducted to conceptually replicate and extend Precursor Study 2 and provide a control condition where we could assess time perception in the absence of political bias. To this end we added a neutral control candidate: Canadian voters read about Tony Abbott – a political leader from Australia - about whom most Canadians were expected to know little and care less. Participants were randomly assigned to read either a (fabricated) negative or positive performance review from six years ago (in 2008; participants were supplied with the objective date). We predicted that supporters of a politician would distance the negative review relative to the positive review and controls, while opponents would distance the positive review relative to the negative review and controls. This study extends Precursor Study 2 both by including a control condition and by including both opposing political candidates (rather than only Obama in Precursor 2).

***Participants and Power***

Participants were recruited from: 1) a local farmers’ market, 2) Canadian political discussion boards on the internet, and 3) Mechanical Turk (selecting for Canadian residents). Ethics approval was obtained from the Wilfrid Laurier University Research Ethics Board (Reference #3953). Participants provided written consent. This process gave an initial sample of 310 participants in total. Because we were examining a motivated memory process, we determined *a priori* to include only those participants who indicated an *in favor* or *opposed* party affiliation on the two central parties represented in our study (Liberal and Conservative). Thus, we did not examine participants who indicated they would vote for another Canadian party). In total, 231 eligible Canadian participants completed the survey (market: 80, discussion boards: 125, Mturk: 26).

Participants were excluded if they failed the manipulation check or stopped the survey before filling out the key DVs. For the same reasons as in the previous two studies, participants who did not indicate affiliation with either the Liberal Party or the Conservative Party were removed from the analyses. As well, participants who were weakly affiliated with their chosen party, such that they gave the lowest response on an affiliation measure, were also excluded. Overall this gave a final sample of 164 participants (99 Liberal, 65 Conservative; 121 male, 42 female, 1 undisclosed; *M*_age_ = 34.15, *SD* = 13.26, Range 18-75). According to G*Power 3.1 (Faul et al., 2009) this sample size was sufficiently sensitive to detect an effect size of *f* = 0.24 (*η_p_²* = 0.05) with 80% power and α = 0.05.

***Procedure***

Participants were invited to participate in a survey on voting strategies in Canada. Market participants completed a pen and paper survey. Internet participants completed the identical survey online.

First, participants indicated the party they would vote for if an election were held today.

The experimental design was a 2 (valence: negative vs. positive) x 3 (political party: Liberal vs. Conservative vs. control) x 2 (voter: Liberal vs Conservative) between-subjects design. Participants read either a *negative* or a *positive* (bogus) Independent Performance Review of one of three political party leaders: (1) the Liberal leader in Canada (Justin Trudeau), (2) the Conservative leader in Canada (Stephen Harper), or (3) neutral: the ‘Labor leader’ in Australia (Tony Abbott); (Abbott is actually the head of a right-wing Liberal party, but to avoid erroneous comparison with Canadian left-wing Liberals, we relabeled him "Labor party"– no participants knew his actual party). The *negative* reviews indicated that an independent Parliamentary Performance Review Committee report was *critical* of [party leader]’s performance in 2008, giving [party leader] a ‘C’ grade for the year 2008. The *positive* reviews indicated that the report *praised* [party leader]’s performance in 2008, giving [party leader] an ‘A’ grade for the year 2008. All participants were fully debriefed after participation, ensuring they understood the performance review was created for the purpose of the study.

After reading the performance review, participants rated the reviews from -3 (*very negative*) to 0 (*neither positive or negative*) to +3 (*very positive*). Next, participants responded to the general personality implicit theories scale. They were then asked to report their level of agreement with three statements designed to capture the degree to which participants felt that the candidate’s past actions should reflect the candidate’s standing in the present. The three items were combined to provide an overall measure of current relevance (α = .77). Finally, on a separate page, participants indicated how distant in time 2008 felt to them (1 = *Almost like yesterday*; 10 = *The distant past*).

**Results and Discussion**

As in Precursor Study 1, participants were included in the current analysis if they intended to vote either Liberal or Conservative and indicated some degree of party affiliation with either the Liberal or Conservative party. This allowed us to examine voters who could clearly be considered motivated *supporters* or *opponents* of the featured candidates. We collected as many participants as possible during the time period leading up to the election, however given the expanded study design we were not able to recruit enough participants to adequately fill all cells. In particular, we recruited somewhat fewer Conservatives, so some “Conservative” cells were low. Due to these issues, small number of participants in some cells when split into all parties (primarily due to difficulty in recruiting enough conservatives), voters were grouped into three conceptually coherent categories for the remainder of the analyses: *supporter* (i.e., defined as Liberal voters in the Liberal candidate condition and Conservative voters in the Conservative candidate condition), *opponent* (e.g., Conservative voters in the Liberal candidate condition and Liberal voters in the Conservative candidate condition), and *neutral* (e.g., both types of voters in the Abbott/neutral condition). We felt this method of simplifying the foregoing analyses was acceptable, as the clear interaction of opposition vs. support is established in Precursor Study 1 and replicated in Precursor Study 2 as well as Studies 1 and 2.

***Subjective Temporal Distance***

We hypothesized that supporters would distance a negative review relative to controls, while opponents would distance the positive review relative to controls. We also predicted that supporters would distance negative reviews relative to positive reviews, and predicted the opposite effect for opponents. A 2 (valence: negative vs. positive) x 3 (voter: supporter vs. opponent vs. neutral) ANOVA with subjective temporal distance as the DV revealed a significant valence by voter interaction; *F*(2, 155) = 6.10, *p* = .003, *η_p_^2^* = = .07.

Opponents rated the positive review as significantly more distant than the negative review, *F*(1, 155) = 11.02, *p* = .001, *η_p_^2^* = = .07. Supporters only rated the negative review marginally more distant that the positive, however, *F*(2, 155) = 2.99, *p* = .086, *η_p_^2^* = = .02. Within control raters, as predicted, there was no difference between condition, *F*(1, 155) = 0.06, *p* = .802, *η_p_^2^* = < .001. Estimated marginal means and standard errors for this analysis can be found in Table S4.

***Path Analysis***

We expected that the interaction between the supporter vs opponent conditions and review valence would predict the subjective temporal distance of the given politician’s performance, which in turn would affect how much participants believed their past performance reflected the present. This was tested using Model 8 in PROCESS [17] with participants in the neutral condition excluded. Review valence was entered as our predictor variable, voter group (supporter vs opponent) as the moderator, subjective temporal distance as the mediator, and perceived relevance as the outcome. The full model is presented in Figure S3. We found that the interaction between voter group and review valence did predict subjective time (a path, ß = -1.35, *t*(99) = -3.52, *p* < .001), but that subjective time did not predict current relevance (b path, ß = -0.13, *t*(98) = -1.22, *p* = .227). Bias corrected bootstrapping with 5,000 samples indicated that our index of moderated mediation was *not* significant (index = 0.17, 95% CI = -0.12, 0.55).

# **Study 1 Additional Information**

**Subjective Time Two-Item Measure**

As in Studies 2 and 5, the original subjective time measure included two slider bars (1 = *Feels very recent*; 100 = *Feels very long ago* and 1 = *Feels like yesterday*; 100 = *Feels like ancient history*, α = .95). Reviewers were concerned that “ancient history” was a loaded endpoint given that the phrase is often used when dismissing past scandals, and we found this issue to be compelling. For this reason, in the main paper we report the results for only the first item (1 = *Feels very recent*). In this section, we will report the results for the two-item measure.

As predicted, there was a significant 3-way interaction; *F*(1, 483) = 38.91, *p* < .001, *η_p_^2^* = .08.

Clinton supporters rated Clinton’s negative incidents as significantly more distant than Trump’s negative incidents, *p* < .001, whereas Clinton’s positive incident was rated significantly closer than Trump’s positive incident, *p* = .002. Furthermore, Clinton’s negative incidents were rated significantly more distant than her positive incident, *p* = .015. Trump’s negative incidents were rated as significantly closer in time than his positive incident, *p* < .001.

Similar patterns emerged for Trump supporters. These supporters rated Trump’s negative incidents as significantly more distant than Clinton’s negative incidents, p < .001, and significantly more distant than Trump’s positive incident, *p* = .002. Trump supporters however rated Clinton’s negative incidents as only marginally closer in time than her positive incident, *p* = .094.

Overall, results support the hypotheses that, regardless of calendar time, partisans distance the failings of their favored candidates and draw close their successes, and conversely perceive their opponents’ missteps as close in time but their successes as more remote.

**Manipulation Check**

Participants who read the negative incidents rated the incidents as significantly worse overall than those who read the positive incidents *F*(1, 483) = 357.51, *p* < .001, *η_p_^2^* = .43. There was also a main effect of candidate *F*(1, 483) = 32.00, *p* < .001, *η_p_^2^* = .06; and a marginal main effect of party affiliation, *F*(1, 483) = 3.81, *p* = .052, *η_p_^2^* = .01; qualified by a candidate × affiliation interaction, where Democrat voters saw a larger gap in valence between Clinton’s events and Trump’s events, versus Republican voters, who saw Clinton’s events and Trump’s events as closer in valence; *F*(1, 483) = 79.59, *p* < .001, *η_p_^2^* = .14. There were no other interactions between valence and candidate or party affiliation (*F*’s < 2.04, *p*’s > .15).

**Comparing Across Events**

In order to examine valence as a factor (by collapsing across the individual negative incidents), we first looked to see if there were any differences in subjective distance ratings (two-item measure) between the negative events for each candidate. We ran separate 2 (voter: Democrat vs. Republican) × 3 (negative incident) ANOVAs. Estimated marginal means and standard errors for this analysis can be found in Table S6.

Among the three Trump negative conditions, a main effect of political party emerged as expected. Trump supporters distanced Trump’s negative incidents relative to Clinton supporters, *F*(1, 156) = 68.13, *p* < .001, *η_p_^2^* = .30. There was no main effect of negative incident, *F*(2, 156) = .95, *p* = .388, or an interaction; *F*(2, 156) = .18, *p* = .836. Indeed, it is notable that although events differed in their time of occurrence by nearly 10 years, no differences emerged in how far events felt.

Similar to the Trump negative conditions, a main effect of political party emerged for the Clinton negative conditions. As expected, Clinton supporters distanced Clinton’s negative incidents relative to Trump supporters, *F*(1, 160) = 10.98, *p* = .001, *η_p_^2^* = .06. Additionally, a main effect of negative incident emerged; *F*(2, 160) = 29.18, *p* < .001, *η_p_^2^* = .27, qualified by a marginal interaction; *F*(2, 160) = 2.88, *p* = .06, *η_p_^2^* = .04. We would expect that all of Clinton’s negative incidents/events would be distanced by Democrat supporters and pulled closer by Republican supporters. However, a closer look at the means for the Clinton negative incidents shows that both Democrat and Republican supporters distanced Clinton’s statement about black youth gangs as super-predators equally. Though we had no a priori expectation of this finding, it is possible that this could represent a boundary condition of distancing, given that this incident was over twenty years old.

Nevertheless, despite this minor anomaly among the negative Clinton events, we opted to collapse across the negative conditions to examine valence (negative vs. positive incidents) as a fixed factor given that any significant findings could be viewed as a more conservative test of our hypothesis.

**Path Analysis**

We expected that the interaction between event valence, candidate, and party allegiance would predict event subjective temporal distance, which in turn would predict how much participants believed the event was relevant to the present. We conducted a test of this relationship by conducting a moderated moderated mediation, using Model 12 of the SPSS macro PROCESS [17], conducted with 5,000 bootstrap samples. Voter intention was entered as our predictor variable, candidate as our first moderator, event valence as our second moderator, subjective temporal distance (two-item) was our mediator, and event relevance was our outcome variable; event date was entered as a covariate to this model. The full model is presented in Figure S4. Standardized z-scores were used for all continuous variables, a practice continued for all path analyses throughout this paper as well as the precursor studies in the addendum. We found that our three-way interaction between voter intention, event candidate, and event valence did indeed predict subjective time (a path, ß = -2.21, *t*(483) = 6.24, *p* < .001), which in turn predicted present relevance (b path, ß = -.19, *t*(482) = -4.70, *p* < .001). Bias-corrected bootstrapping indicated that our index of moderated moderated mediation was significant (index = 0.43, 95% CI = 0.20, 0.72) again suggesting that part of the effect of the interaction on beliefs about current relevance occurs through how far in the past political events feel. When Clinton voters were viewing Clinton events, negative events felt farther than positive events, which in turn made them feel less relevant (effect = -0.07, 95% CI = -0.14, -0.02). When they were viewing Trump events, there was no significant indirect effect (effect = 0.07, 95% CI = -0.02, 0.17). When Trump voters were viewing Clinton events, negative events felt closer than positive events, which in turn made them feel *more* relevant (effect = 0.17, 95% CI = 0.07, 0.29). When they were viewing Trump events, negative events felt farther than positive events, which in turn made them feel *less* relevant (effect = -0.12, 95% CI = -0.24, -0.04).

# **Study 2 Additional Information**

**Subjective Time Two-Item Measure**

As in Studies 1 and 5, the original subjective time measure included two slider bars (1 = *Feels very recent*; 100 = *Feels very long ago* and 1 = *Feels like yesterday*; 100 = *Feels like ancient history*, α = .94). Reviewers were concerned that “ancient history” was a loaded endpoint given that the phrase is often used when dismissing past scandals, and we found this issue to be compelling. For this reason, in the main paper we report the results for only the first item (1 = *Feels very recent*). In this section, we will report the results for the two-item measure.

We again hypothesized that opponents of the candidates would distance positive events relative to supporters, while the reverse would occur for negative events. However, because an election victory is a positive event for supporters, but a negative event for opponents, we predicted that a positive election passage would be a positive event for supporters but a negative one for opponents, and that the opposite would be true of the negatively valenced election passage. Thus, we hypothesized that Clinton supporters would perceive the positively valenced election passage as a negative event, and distance it accordingly, relative to Trump supporters, who would perceive the election more positively. When Trump’s victory was portrayed as more questionable, and thus in a more negative light, however, we expected this pattern to reverse.

We conducted a 3 (valence: positive vs. negative vs. control) x 2 (voting preference: Clinton vs. Trump) ANOVA with subjective temporal distance from Election Day 2016 as the DV. Estimated marginal means and standard errors for this analysis can be found in Table 2. As predicted, we found a significant 2-way interaction between valence and voting preference, *F*(2, 321) = 4.31, *p* = .014, *η_p_^2^* = .03; there was no main effect of valence or of voting preference.

Within the negatively valenced election description condition, Clinton supporters indicated that the original election felt significantly closer to present than did Trump supporters, p = .029, while in the positively valenced condition, Trump supporters rated the election as feeling marginally closer to present than did Clinton supporters, p = .051. There was no significant difference in the control condition, *p* = .745.

Differences were primarily found among Clinton supporters, who indicated that the positively valenced election passage felt significantly more distant than the negative passage, *p* = .012. The positive passage also felt marginally further than in the control condition, *p* = .057. For Trump supporters, the negative passage felt marginally further than the positive one, *p* = .082.

**Additional Measures**

Two questions asked, on an eleven-point scale, their level of surprise at the election’s outcome (1 = *Not at all surprised*; 11 = *Extremely surprised*), and their level of pleasure about the election’s outcome (1 = *Extremely displeased*; 11 = *Extremely pleased*). Finally, we assessed agreement with the following statement: “Whether they voted for him or not, people should recognize that Donald Trump is legitimately President of the United States of America” (1 = *Strongly disagree*; 100 = *Strongly agree*).

In addition to our key dependent variables, we included several other measures. Two questions asked about media consumption, with one asking how much participants followed the news *during* the 2016 election, and one asking how much participants have followed the news *since* the election (1 = *Not at all*, 7 = *A great deal*).

**Other Mechanisms for Subjective Temporal Distance From Election**

We expected to find that a framing by voter affiliation interaction would be the best predictor of subjective distance from the 2016 US presidential election. We know, however, that other factors can influence perceptions of subjective time from an event, such as greater emotion [12] and fluency [18]. Thus, we tested to see if our measure of subjective distance (two-item measure) could be predicted by either emotional, affiliative factors (surprise at election results, pleasure about election results, feelings about presidential legitimacy), or by cognitive, event fluency factors (exposure to news both during and after the election). Correlation coefficients for this analysis can be found in Table S8. We found that despite the fact that the three emotional factors were significantly related to voter affiliation, to the other measures of subjective time, and to one another, they did not significantly predict perceptions of subjective distance from the 2016 election. Fluency, via degree of news exposure after the election, was unrelated to subjective distance, though it was marginally correlated with perceptions of legitimacy; degree of news exposure during the election was marginally related to feeling subjectively closer to the election, though entering that variable as a covariate in the primary analysis had no impact on the initial findings. Likewise, controlling for post-election news exposure, or any of the emotional factors, did not affect the original pattern of results.

Because of the large degree of media exposure reported by our participants and its possible connection to perceptions of legitimacy, we conducted one additional exploratory analysis. We conducted a post-election media exposure × voter affiliation regression on the legitimacy item. We discovered a main effect of voter affiliation (*b* = 20.95, β = .59, *p* < .001, 95% confidence interval [CI] = [17.89, 23.96]), and no effect of news consumption (*b* = -.62, β = -.03, *p* = .556 , 95% CI = [-2.67, 1.44]). This was qualified by a significant interaction (*b* = 3.20, β = .51 *p* = .002 , 95% CI = [1.15, 5.25]), suggesting that voter affiliation moderates the effect of news exposure on perceptions of legitimacy. Simple slopes analyses for the effect of reported voter affiliation at one standard deviation above and below the mean for the post-election news consumption variable, showed that while voter affiliation significantly predicted perceptions of legitimacy regardless of level of news consumption, the effect was smallest for those who reported the least news exposure (*b* = 15.93, , β = .46 *p* < .001 , 95% CI = [11.53, 20.33]), and largest for those with the *most* news exposure (*b* = 25.96, , β = .75 *p* < .001 , 95% CI = [21.48, 30.43]). Across voter affiliation, Clinton voters felt that the Trump presidency was less legitimate given more news exposure; *b* = -3.81, β = -.17, *p* = .001, 95% CI = [-6.14, -1.49]. In contrast, Trump voters felt that the Trump presidency was more legitimate given more news exposure, though this failed to reach significance (*b* = 2.58, β = .12, *p* = .134, 95% CI = [-.05, .27]). It is worth noting, however, an immense ceiling effect among Trump affiliated voters; those who had little news exposure had a mean score of 90.63 of 100 on the legitimacy scale, while those with high news exposure had a mean score of 98.37

# **Study 3 Additional Information**

**Manipulation Check**

Those reading a negative review perceived it as more negative than those reading a positive review, *F*(1,448) = 1505.93, *p* < .001, *η_p_^2^* = .77. There was also a main effect of voter type, *F*(2,448) = 4.53, *p* = .011, *η_p_^2^* = .02. Opponents rated the performance reviews as more negative on average than supporters, *p* = .005, and neutral raters, *p* = .021. Supporters and neutrals did not differ from one another, *p* = .569.

**Path Analysis**

At the study’s outset, we devised a multi-step serial mediation process. We examined how voter group (opponent vs supporter) would predict how far away the success/failure felt, which would in turn predict perceived relevance of the information, which would then predict candidate perception, which would then predict voting intention. Furthermore, we expected this path to be different when the politician review was positive rather than negative. Not only should opponents be more motivated to distance a review of the opposing politician when it was positive rather than negative (and supporters vice versa), but the current relevance of the review should be related to opposite effects on the character judgment (person perception) of the politician depending on valence as well. This was tested using Model 6 in PROCESS [17] with participants in the neutral condition excluded and separate analyses run for the positive and negative valence conditions. Voter group (supporter vs opponent) was entered as our predictor variable; subjective temporal distance (two-item), current relevance, and person perception as the first, second, and third mediator respectively; and voting intention as the outcome.

For the positive review, we found that voter group did not predict subjective time (a path, ß = -0.06, *t*(132) = -0.36, *p* = .720), and that subjective time did not predict current relevance (b path, ß = -0.08, *t*(131) = -0.94, *p* = 351). However, current relevance did predict person perception (c path, ß = 0.37, *t*(130) = 5.85, *p* < .001), and person perception did predict voting intention (d path, ß = 0.68, *t*(129) = 9.12, *p* < .001). Bias corrected bootstrapping with 5000 samples indicated that overall our indirect path was not significant 95% CI [-0.01, 0.01]. The path model for the positive review condition is presented in Figure S5.

For the negative review, we found that voter group predicted subjective time (a path, ß = 0.63, *t*(138) = 4.02, *p* < .001), which in turn predicted current relevance (b path, ß = -0.31, *t*(137) = -3.43, *p* < .001), which in turn predicted person perception (c path, ß = -0.50, *t*(136) = -9.64, *p* < .001), and character judgement did predict voting intention (d path, ß = 0.52, *t*(135) = 7.53) *p* < .001). Bias corrected bootstrapping with 5000 samples indicated that our indirect path was significant, 95% CI [0.01, 0.11]. The path model for the negative review condition is presented in Figure S6.

# **Study 4 Additional Information**

**Path Analysis**

We examined if the manipulated time condition would predict the subjective temporal distance, which would in turn predict candidate perception, which would in turn predict voting intention. This was tested using Model 6 in PROCESS [17]. Time condition was entered as our predictor variable with participants in the neutral condition excluded; subjective temporal distance (two-item), current relevance, and person perception as our first, second, and third mediators respectively; and voting intention as our outcome. Gender and voting group were included as covariates. The full path model is presented in Figure 6. We found that time condition predicted subjective time (a path, ß = 0.38, *t*(256) = 3.06, *p* = .002), which in turn predicted current relevance (b path, ß = -0.55, *t*(255) = -10.18, *p* < .001), which in turn predicted person perception (c path, ß = -0.36, *t*(254) = -5.76, *p* < .001), which in turn predicted voting intention (d path, ß = 0.58, *t*(253) = 10.92, *p* < .001). Bias corrected bootstrapping with 5000 samples indicated that our mediation pathway was significant, 95% CI [ 0.01, 0.08]. That is, participants in the distant condition compared to the close condition saw Bosch’s infidelity as further away, which in turn made it seem less relevant, which then made him perceived more favorably, which then made them more likely to say they would vote for him.

# **Study 5 Additional Information**

**Notes**

A mistake in the preregistration of Study 5 stated that participants would be recruited from Crowdflower, when in fact we recruited from CloudResearch.

**Subjective Time Two-Item Measure**

As in Studies 1 and 2, the original subjective time measure included two slider bars (1 = *Feels very recent*; 100 = *Feels very long ago* and 1 = *Feels like yesterday*; 100 = *Feels like ancient history*, α = .95). Reviewers were concerned that “ancient history” was a loaded endpoint given that the phrase is often used when dismissing past scandals, and we found this issue to be compelling. For this reason, in the main paper we report the results for only the first item (1 = *Feels very recent*). In this section, we will report the results for the two-item measure.

As predicted, Republicans saw that Capitol attack as significantly farther away than Democrats, *F*(1, 701) = 76.44, *p* < .001, *η_p_^2^* = .098. There was also a marginal main effect of focus condition, such that the attack seemed somewhat closer in the Republican focus condition than the fringe condition, *F*(1, 701) = 3.10, *p* = .079, *η_p_^2^* = .004. Contrary to our predictions, however, there was no interaction between voter affiliation and focus condition, *F*(1, 701) = 0.08, *p* = .773, *η_p_^2^* < .001. Estimated marginal means and standard errors can be found in Table 9. The lack of interaction is inconsistent with our prediction that Republican participants would distance the Capitol attack more when it was closely tied to the Republican party (vs a fringe), whereas Democrats might view it as closer when it was more linked to Republicans. As noted in the prior section, it may be that the overwhelming attribution for blame to the fringe (and other sources) across both focus conditions weakened the impact of any perceived responsibility of Republican voters and leaders and diminished the chance of detecting a condition effect. It may also be that both attributions to Republicans and to a distasteful fringe are equally threatening to Republicans and motivate the same desire to distance regardless of the frame.

**Additional Measures**

Participants were asked how much responsibility for the storming of the US Capitol they would assign to a variety of different entities on a scale of 1 = *No Responsibility* to 10 = *Complete Responsibility*. The entities rated included extremist fringe groups, extremist individuals, Democrat political leaders, regular Democrat Voters, Republican political leaders, regular Republican voters, the mainstream media, social media, and misinformation.

Participants were asked to rate the morality of several of the relevant groups on a scale of 1 = *Very Immoral* to 7 = *Very Moral*. The groups included were the Republican Party, Republican voters, the Democratic Party, Democrat voters, and Donald Trump.

Attitudes “towards the Capitol storming” were assessed using six items asking how moral, shameful, justified, harmful, violent, and serious they found the event to be, ranging from 1 (e.g., *Very Immoral*) to 7 (e.g., *Very Moral*). The items demonstrated good reliability (α = .93) and were aggregated such that higher scores represented more negative reactions.

Several measures assessed group identity. There were two single-item measures asking participants to rate the strength of their political affiliation from 1 = *Not affiliated at all* to 7 = *Highly affiliated* and how much pride they felt in their political party from 1 = *None at all* to 7 = *A lot*, respectively, *r*_SB_ = .81. We also included the Verbal Fusion Scale [33] as a means of measuring party identification. See below for details on the measure and analyses examining it as a potential moderator.

**Attitudes Regarding the Capitol Storming**

While participants in both political parties saw the Capitol attack as generally negative, Republican participants (*M* = 5.32, *SE* = 0.06) held significantly less negative attitudes about the event compared to Democrats (*M* = 6.65, *SE* = 0.06), *F*(1, 703) = 227.92, *p* < .001, *η_p_^2^* = .245 (higher scores mean more negative reactions). There was not, however, any difference between the Republican focus condition focus (*M* = 6.00, *SE* = 0.06) and fringe focus condition (*M* = 5.97, *SE* = 0.06), *F*(1, 703) = 0.10, *p* = .749, *η_p_^2^* < .001, nor was there any interaction between voter affiliation and focus condition, *F*(1, 703) = 0.03, *p* = .872, *η_p_^2^* < .001.

**Assigned Responsibility**

The intention of the focus manipulation was to heighten or reduce the degree to which Republicans (voters and leaders) were deemed responsible for the storming of the Capitol. Responsibility ratings can be found in Table S10 (inferential statistics) and Table S11 (means and standard errors).

Overall, results indicate that the focus condition significantly shifted people’s views of responsibility for the incident, but only to a limited degree. Republican voters were viewed as more responsible in the Republican focus condition, and Republican leaders were seen as more responsible in that condition but only among Republican participants. In contrast, the focus manipulation did not affect people’s judgements of the responsibility of fringe groups. Although this manipulation check revealed some effect of focus condition, a more thorough examination of responsibility ratings suggest some reason for caution. First, Republicans downplayed the responsibility of Republican voters and leaders across both parties: relative to Democrats, and relative to other sources of responsibility, Republicans gave their own ingroup a pass, blaming fringe groups, media, social media, misinformation, and even Democrats as more responsible than Republicans for the incident across both focus conditions. As a result, although there was a significant increase in responsibility of Republicans caused by the focus condition, it may not be a *psychologically meaningful* increase – especially among Republican participants that the focus condition was especially intended to target - due to all the other sources of blame.

**Path Analysis**

We examined if voter affiliation would predict subjective temporal distance from the Capitol storming, which would in turn predict people’s perceptions of how relevant the event was overall, which would in turn predict moral ratings of the Republican party. We conducted a test of this relationship by conducting a serial mediation using Model 6 in PROCESS [17], conducted with 5,000 bootstrap samples. Voter affiliation was entered as our predictor variable, subjective time (two-item) as our first mediator, current relevance as our second mediator, and Republican party morality as our outcome variable. The full model is present in Figure S8. We found that voter affiliation on its own *did* predict subjective time (a path, ß = -0.62, *t*(703) = -8.66, *p* < .001), which in turn predicted current relevance, (b path, ß = -0.24, *t*(702) = -9.23, *p* < .001), which in turn predicted Republican party morality (c path, ß = -0.34, *t*(701) = -9.49, *p* < .001). This time the overall indirect effect was significant (effect = -0.05, 95% CI = -0.07, -0.03).

We also ran a version of the path analyses with focus condition added to the model. In a custom model in PROCESS [17], focus condition was entered as our predictor variable, voter affiliation as our moderator, subjective time as our first mediator, current relevance to the Republican party as our second mediator, and Republican party morality as our outcome variable. We found that the interaction between voter affiliation and focus condition did *not* predict subjective time, (*a* = -0.02, *t*(701) = -0.29, *p* = .773). Subjective distance did, however, predict current relevance (*b* = -0.44, *t*(702) = -12.97, *p* < .001), which in turn predicted Republican party morality (*c* = -0.35, *t*(699) = -9.55, *p* < .001). Bias-corrected bootstrapping indicated that our index of moderated mediation was *not* significant (index = -0.003, 95% CI = -0.02, 0.02). We also examined the conditional indirect effects. There were no significant indirect effects for Republicans (effect = -0.01, 95% CI = -0.03, 0.01) or Democrats (effect = -0.01, 95% CI = -0.03, 0.002).

**Group Identity**

Though our focus manipulation was ineffective at shaping how participants shifted their perception of time and current relevance, our affiliation strength/party pride variable provided an alternate means of assessing how motivation to protect one’s group could moderate the relationship between voter affiliation and subjective time (the two-item measure) as well as current relevance. Though not part of our main hypotheses, we considered that stronger affiliation/pride could predict more subjective time and less relevance for Republicans and less subjective time and more relevance for Democrats. Our analyses included two steps. First, the measure would be tested as a dependent variable with voter affiliation and focus condition as predictors. If affiliation strength/ party pride did not vary based on focus condition (or a voter affiliation × focus condition interaction) we would proceed to the second step, in which the measure was tested as a moderator of the relationships between voter affiliation and subjective time, and voter affiliation and current relevance.

With our combined affiliation strength/party pride measure as the dependent variable, there was no main effect of focus condition, *F*(1, 703) = 0.86, *p* = .354, *η_p_^2^* = .001, nor was there a main effect of voter affiliation, *F*(1, 703) = 0.40, *p* = .525, *η_p_^2^* = .001, or an interaction, *F*(1, 703) = 0.56, *p* = .456, *η_p_^2^* = .001. For this reason, we proceeded to test the affiliation strength/party pride measure as a moderator.

We ran a simple moderation analysis in PROCESS (Hayes, 2017) with voter affiliation and the standardized affiliation strength/party pride scores as predictors and the standardized subjective time measure as the outcome variable. Republicans saw the Capitol storming as farther away than Democrats, *ß* = -0.62, *t*(701) = -8.68, *p* < .001. There was also a main effect of affiliation strength/party pride, with higher affiliation/pride associated with more subjective temporal distance, *ß* = 0.26, *t*(701) = 2.29, *p* = .022. The main effects were qualified by the predicted interaction between voter affiliation and affiliation strength/party pride, *ß* = -0.18, *t*(701) = -2.46, *p* = .014. For Republicans, more affiliation strength/party pride was marginally associated with more subjective distance, *ß* = 0.09, *t*(701) = 1.67, *p* = .095, while for Democrats, more affiliation strength/party pride was associated with *less* subjective distance, *ß* = -0.09, *t*(701) = -1.80, *p* = .072. The pattern of results is depicted in Figure S9.

We ran an otherwise identical model with current relevance as the outcome variable. Republicans saw the storming as less relevant than Democrats, *ß* = 1.46, *t*(703) = 29.36, *p* < .001, while affiliation strength/party pride was associated with less relevance, *ß* = -0.58, *t*(703) = -7.24, *p* < .001. There was again as significant interaction between voter affiliation and affiliation strength/party pride, *ß* = 0.34, *t*(703) = 6.88, *p* < .001. For Republicans, more affiliation strength/party pride was associated with less current relevance, *ß* = -0.24, *t*(703) = -6.54, *p* < .001, while for Democrats, more affiliation strength/party pride was associated with *more* relevance, *ß* = 0.11, *t*(703) = 3.10, *p* < .001. The pattern of results is depicted in Figure S10. Results for both subjective time and current relevance do not change if focus condition is added to the model.

In addition to the affiliation strength and party pride measures, we also included the Verbal Fusion scale (Gomez et al., 2011), which included seven items rated from 1 = *Strongly Agree* to 7 = *Strongly Disagree* based on how “fused” the participant felt with their political party (e.g. “I am one with the Republican Party.”, “I have a strong emotional bond with the Democratic Party.”). Items were automatically modified to match the participants’ self-identified preferred party. Though the scale showed good reliability (α = .95), a note of caution is warranted: by error the endpoints were in the reverse direction of most measures in the survey (with *Strongly Agree* as the lowest option and *Strongly Disagree* as the highest). Because all other scales had endpoints ordered in the opposite direction, even mildly inattentive participants might have responded without checking endpoints carefully enough. Another concern was that the verbal fusion scale was not strongly related with the other measures. While the affiliation strength and party pride measures were correlated at *r* = .68, verbal fusion was much more weakly correlated to both affiliation strength, *r* = .19, and party pride, *r* = .20. We therefore focus on the affiliation strength and party pride measures, which we combined into a single measure that showed good reliability (*r_SB_* = .81).

Along with the moderation analyses involving the affiliation strength/group pride variable, we also ran an equivalent set of analyses with the verbal fusion measure (Gomez et al., 2011), which is reported in the supplement due to our low confidence in the measure in the context of our study. As with the affiliation/pride variable, we begin by testing verbal fusion as a dependent variable, then move on to testing it as a moderator.

With verbal fusion as a dependent variable, there was no main effect of focus condition, *F*(1, 703) = 0.41, *p* = .521, *η_p_^2^* = .001, or any interaction between focus condition and voter affiliation, *F*(1, 703) = 1.78, *p* = .183, *η_p_^2^* = .003. There was a marginal effect of voter affiliation, such that Republicans reported more fusion than Democrats, *F*(1, 703) = 3.21, *p* = .079, *η_p_^2^* = .005. These results allowed us to proceed to the next step and test verbal fusion as a moderator.

As with affiliation strength/party pride, we ran a model with voter affiliation and the standardized verbal fusion scores as predictors and the standardized subjective time measure as the outcome variable. There was a main effect of voter affiliation, such that Republicans saw the Capitol storming as more distant in time, *ß* = -0.63, *t*(701) = -8.70, *p* < .001, but there was no main effect of verbal fusion, *ß* = -0.01, *t*(701) = -0.07, *p* = .944, or interaction, *ß* = -0.02, *t*(701) = -0.28, *p* = .800.

The same moderation model was run with current relevance as the outcome variable. Again there was a main effect of voter affiliation with Republicans seeing the storming as less relevant than Democrats, *ß* = 1.46, *t*(703) = 28.32, *p* < .001, but there was no main effect of fusion, *ß* = -0.11, *t*(703) = -1.37, *p* = .170, or interaction, *ß* = 0.05, *t*(703) = 1.07, *p* = .287.

It is unclear why verbal fusion did not act as a moderator in the same fashion as our affiliation strength/party pride variable, though it may be due to how the scale endpoints were reversed from most other measures in the survey, as mentioned above. This may have introduced additional error into the analyses and dampened the effect. Though there could be something about fusion that makes it act differently in this scenario, it is not obvious what that would be. It is possible that the moderating effect of group identity is simply not strong enough to appear consistently.

# **Supporting Tables**

## **Table S1. Quotes taken from Liberal and Conservative Candidates from around the year 2000. (Precursor Study 1)**

| Liberal Candidate (Michael Ignatieff) | Conservative Leader (Stephen Harper) |
| --- | --- |
| “If I am not elected (in Canada), I imagine I will ask Harvard to take me back,” Ignatieff said. “I hope I’ll be back in some shape or form.”  —Michael Ignatieff, *Harvard Crimson*  Michael Ignatieff referred to the UK as his “adopted country,” and voted in their elections instead of Canada’s.  —Michael Ignatieff, *Blood and Belonging*  Ignatieff called the Canadian flag a “passing imitation of a beer label.”  —Michael Ignatieff, *Observer Magazine* (UK)  “You have to decide what kind of America you want….It’s your country, just as it is mine.”  —Michael Ignatieff while working in the United States, CPSAN  Ignatieff said that Canada has an entirely “bogus” reputation for being peacekeepers. He went on, saying “we used to have this ability, but we gave it away.”  —Michael Ignatieff, lecture at Trinity College. | “In terms of the unemployed, of which we have over a million-and-a-half, I don’t feel particularly bad for many of these people.”  —Stephen Harper, speaking in Montréal  “Canada appears content to become a second-tier socialistic country, boasting ever more loudly about its economy and social services to mask its second-rate status.”  —Stephen Harper, *National Post*  “If you’re like all Americans, you know almost nothing except for your own country. Which makes you probably knowledgeable about one more country than most Canadians.”  —Stephen Harper, addressing an American Republican lobby group  “Canada is a Northern European welfare state in the worst sense of the term, and very proud of it.”  —Stephen Harper, addressing an American Republican lobby group  “The is no upside to the position Canada took.”  —Stephen Harper on Canada refusing to join the United States’ war on Iraq as part of the “Coalition of the Willing,” *Maclean’s* |

## **Table S2. Estimated marginal means and standard errors by cell for subjective temporal distance. (Precursor Study 1)**

|  | Conservative Leader | Liberal Leader | Total |
| --- | --- | --- | --- |
| Conservative Voters | 6.81  (0.44) | 4.80  (0.46) | 5.81  (0.32) |
| Liberal Voters | 4.39  (0.49) | 6.97  (0.40) | 6.18  (0.32) |
| Total | 3.60  (0.33) | 6.39  (0.30) | 5.99  (0.22) |

Numbers in parentheses correspond to standard errors.

## **Table S3. Estimated marginal means and standard errors by cell for subjective temporal distance. (Precursor Study 2)**

|  | Positive Evaluation | Negative Evaluation | Total |
| --- | --- | --- | --- |
| Democrats | 54.87  (2.88) | 64.06  (3.11) | 59.46  (2.12) |
| Republicans | 66.71  (3.92) | 42.53  (4.04) | 54.62  (2.82) |
| Total | 60.79  (2.43) | 53.29  (2.55) | 57.04  (1.76) |

Numbers in parentheses correspond to standard errors.

## **Table S4. Estimated marginal means and standard errors by cell for subjective temporal distance. (Precursor Study 3)**

|  | Positive Evaluation | Negative Evaluation | Total |
| --- | --- | --- | --- |
| Supporter | 6.13  (0.62) | 7.48  (0.48) | 6.81  (0.39) |
| Opponent | 7.16  (0.39) | 5.12  (0.48) | 6.14  (0.31) |
| Neutral | 5.80  (0.44) | 5.64  (0.45) | 5.72  (0.31) |
| Total | 6.36  (0.28) | 6.08  (0.27) | 6.22  (0.20) |

Numbers in parentheses correspond to standard errors.

## **Table S5. Past incidents involving Hillary Clinton and Donald Trump. (Study 1)**

| Democrat Candidate (Hillary Clinton) | Republican Candidate (Donald Trump) |
| --- | --- |
| **POSITIVE INCIDENT** | |
| The actions described spanned from **2001 to 2004**.    Hillary Clinton was New York Senator at the time of the September 11, 2001 attacks. She subsequently took a leading role in investigating the health issues faced by 9/11 first responders.    Hillary Clinton stood out particularly for her work on legislation addressing the health needs of first responders who’d spent days or weeks breathing in a toxic cloud of PCB’s and other chemicals in Lower Manhattan.    Firefighter Richard Alles said what struck him most about Clinton, he said, was what he called her “compassion”. “She really went out of her way to speak to the first responders on the site to reassure them,” he said. “I never forgot it.”    Alles also noted how Clinton quickly grasped the potential health risks of Ground Zero, and how doggedly she pursued treatment for those who suffered. “We all knew from the get-go that the air was contaminated, but we had a job to do so we kept on working. Sen. Clinton was at the forefront over dealing with it, she showed herself to be a fighter.”    Andrea Bernstein of WNYC radio described Hillary Clinton’s reaction upon discovering that “the Bush administration had instructed officials of the federal Environmental Protection Agency to reassure New Yorkers after 9/11 that the air over Ground Zero was safe. In fact, they had a pretty good idea that it was a toxic pall of asbestos, cement, glass dust, heavy metals, fuels and PCBs.” Clinton was livid. “I don’t think any of us expected that our government would knowingly deceive us about something as sacred as the air we breathe,” Clinton said, her voice tightening in anger. “The air that our children breathe in schools, that our valiant first responders were facing on the pile [of Ground Zero rubble].”    “I am outraged,” Clinton went on. “In the immediate aftermath, the first couple of days, nobody could know. But a week later? Two weeks later? Two months later? Six months later? Give me a break!”    Within weeks of the attacks, she had helped secure $12 million for a pilot project screening approximately 9,000 workers with suspected Ground Zero illnesses. By April 2004 the program had grown to a $90 million fund, which offered three free medical exams a year to 50,000 first responders and Lower Manhattan residents.    In 2004, first responders held a ceremony for Clinton, thanking her for the passage of the health care legislation. “Senator Clinton, thank you very much, if you decide to throw your hat in the ring to run for the other office, you have my support,” said Chuck Capo, head of EMS local 3621. | The actions described spanned from **2002 to 2005**.  In 2002, Mark Burnett, creator of the popular reality television series “Survivor,” visited Donald Trump, a well-known real estate magnate and businessman, at Trump Tower. He pitched “The Apprentice” – which would star Trump as judge, jury and executioner in fierce competition between young, ambitious hopefuls. As Mark Fisher reports in the Washington Post, Trump’s agent told him it was a terrible idea — business shows never work on TV. Trump disagreed, and fired the agent shortly thereafter. “If I would have listened to him,” Trump told The Washington Post, “I wouldn’t have done the show.” At that meeting, Trump secured not only a starring role on a show made by TV’s hottest producer but also a 50 percent ownership stake. He made the decision on the spot, Fisher says, “It was a classic Trump moment, an example of [his] gut-instinct decision-making.” Trump later reported that he earned about 15 million per season on the show.  Trump took to his role instantly. “It was only when we saw rough cuts of the boardroom scenes that we realized they were gold,” Jeff Gaspin, NBC head of reality shows, said. “After the first episode, we said we want more Trump.”  The Apprentice premiered on NBC in 2004 to great ratings. The season started with 16 contestants, with one contestant eliminated, or “fired” by Trump at the conclusion of each week's episode. Each year, the winning contestant earns a spot as Trump’s apprentice for a year, which comes with a $250,000 payday.  “Donald was about honesty. He was tough but truthful,” said Gaspin. “He wasn’t saying you were good at your job when you weren’t.”  In its first season it was the 7th most watched show on television with 21 million viewers per week. This made it the most popular new show of 2004. The show earned Trump widespread exposure and celebrity, and even a star on the Hollywood Walk of Fame.  Bill Rancic, the winner of The Apprentice's first and most watched season, told Fortune that Trump is a "guy who held up his end of the bargain." Rancic added, "He truly took me under his wing."  An entrepreneur in his own right, Rancic spoke fondly about having the opportunity to watch Trump negotiate deals during his apprenticeship following the show. During his stint with Trump, he helped lead the construction of the iconic Trump Tower Chicago - one of the tallest skyscrapers in the world.  Rancic said he learned another important lesson from Trump: "You've got to have good people around you," he said. "You can't do it alone." Rancic continued, "He knows how to get the best out of those people." |
| **NEGATIVE INCIDENT** | |
| **Superpredators**  In a 1994 C-SPAN recording, Hillary Clinton stated:  "…we also have to have an organized effort against gangs," she said. "Just as in a previous generation we had an organized effort against the mob. We need to take these people on. They are often connected to big drug cartels, they are not just gangs of kids anymore. They are often the kinds of kids that are called superpredators — no conscience, no empathy. We can talk about why they ended up that way, but first, we have to bring them to heel."  This statement has come to be widely criticized as labeling inner city youths (mainly young black men) “superpredators.” | **Groping**  In 2005, Donald Trump was recorded in a “hot mic" conversation with Billy Bush of Access Hollywood.  “You know I’m automatically attracted to beautiful [women]— I just start kissing them. It’s like a magnet. Just kiss. I don’t even wait.  And when you’re a star they let you do it. You can do anything…  Grab them by the p**** [genitals]. You can do anything.”  This statement has come to be widely criticized as condoning sexual assault. |
| **Emails**  In 2009, Hillary Clinton became Secretary of State and began using an email account housed on a private server. At the time, the State Department's policy stated that "normal day-to-day operations" were to be conducted on an authorized system. When this practice later came to light in 2014, an F.B.I. investigation into recommended no charges against Hillary Clinton but called her use of email "extremely careless." | **McCain POW**  In July 2015, Donald Trump said of former Republican nominee for president and Vietnam prisoner of war John McCain, “He’s not a war hero… He’s a war hero because he was captured. I like people that weren’t captured, OK?” |
| **Iraq**  In 2002, Hillary Clinton, then New York Senator, voted in favor of the Resolution authorizing the use of military force against Iraq. More than a year after the U.S. invasion and the Bush administration's acknowledgement that Iraq did not have Weapons of Mass Destruction or ties to Al-Qaeda, Clinton stated it was “the right vote,” adding “I don’t regret giving the president authority.” This decision to support the Iraq war has come to be widely criticized as the motives and outcomes of the War have been seriously questioned. | **Bankruptcy**  In 2009, Trump Entertainment Resorts went bankrupt. In an interview with Chris Wallace from Fox News, Wallace asked: "In that case alone lenders to your company lost over a billion dollars and over 1,100 people were laid off. Is that the way that you'd run this country?  Donald Trump: "Let me tell you about the lenders. First of all these lenders aren't babies. These are total killers. These are not the nice sweet little people that you think, OK? You know. You are living in a world of the make-believe Chris if you want to know the truth."  Trump concludes his debate answer with this: Donald Trump: "...I made a lot of money in Atlantic City and I'm very proud of it I want to tell you that. Very very proud of it."  These actions have come to be widely criticized as Trump benefited financially while many employees, contractors and creditors suffered. |

Item descriptor is as follows, *“We are interested in people’s reactions to the actions of public figures that occurred in the past. We will ask you to consider one such past incident.”*

## **Table S6. Estimated marginal means and standard errors by cell for subjective temporal distance in preliminary analyses. (Study 1)**

|  | Trump Negative Incidents | | | |
| --- | --- | --- | --- | --- |
|  | Groping | McCain POW | Bankruptcy | Total |
| Clinton Voters | 23.45  (4.07) | 31.91  (4.25) | 31.73  (4.31) | 6.26  (0.14) |
| Trumps Voters | 61.42  (5.06) | 65.40  (6.40) | 64.18  (5.68) | 68.34  (3.46) |
| Total | 42.43  (3.25) | 48.66  (3.84) | 47.96  (3.57) | 51.49  (2.32) |
|  | | | | |
|  | Clinton Negative Incidents | | | |
|  | Superpredators | Emails | Iraq | Total |
| Clinton Voters | 67.02  (4.38) | 42.86  (4.07) | 61.96  (4.25) | 54.25  (2.54) |
| Trump Voters | 66.78  (4.95) | 22.06  (5.84) | 46.02  (5.54) | 43.28  (3.28) |
| Total | 66.90  (3.31) | 32.46  (3.56) | 53.99  (3.49) | 48.77  (2.12) |

Numbers in parentheses correspond to standard errors.

## **Table S7. Descriptions of 2016 election. (Study 2)**

| Positive Framing | Negative Framing |
| --- | --- |
| On Nov. 8 2016, Donald Trump was elected the 45^th^ President of the United States of America. His unexpected and dramatic win was historic in many ways. Although almost all polls and experts predicted he would lose, he surprised the world with a victory. Trump was a political novice who flouted convention and who was critical of “business as usual” political corruption and elites on both sides of the political divide. His promise to “drain the swamp” of elites, corruption, and big money lobbyists resonated with many voters. Many felt that his win signaled a turning point in which Americans’ displeasure with a dysfunctional political system was finally heard. Following his victory, Trump said “I pledge to every citizen of our land that I will be president for all Americans,” and “For those who have chosen not to support me in the past, of which there were a few people, I’m reaching out to you for your guidance and your help so we can work together and unify our great country.” | On Nov. 8 2016, Donald Trump was elected the 45th President of the United States of America. His unexpected and dramatic win was historic in many ways. Although almost all polls and experts predicted he would lose, he surprised the world with a victory. Trump was a political novice who flouted convention, and gained popularity with several segments of the American population. However, the decisiveness of his victory has been contested on a couple of key grounds. First, although Trump won the electoral college vote, Clinton won the popular vote by more than 2 million votes, making him the 5th US president to win an election while losing the popular vote. Further, serious concerns have been raised about Russian interference in the election. Although little evidence suggests direct vote machine interference, mounting evidence suggests that Russia played a role in acquiring the Democratic National Committee’s emails and that they deliberately spread misleading news and memes via social media (for instance purchasing advertising on Facebook), and in contributing to polarized social discourse by using “trolls” and “bots” to impersonate Americans in online discussion. |

## **Table S8. Correlations between types of subjective distance, emotion and fluency variables, and vote support.**

|  | | | | | | | | |
| --- | --- | --- | --- | --- | --- | --- | --- | --- |
| Measure | **1** | **2** | **3** | **4** | **5** | **6** | **7** | **8** |
| 1. Subjective temporal distance from 2016 election | – |  |  |  |  |  |  |  |
| 2. Subjective temporal length of presidency | .48^***^ | – |  |  |  |  |  |  |
| 3. Subjective temporal distance from 2020 election | .11^*^ | .22^***^ | – |  |  |  |  |  |
| 4. Surprised by 2016 election outcome | -.05 | .21^***^ | .30^***^ | – |  |  |  |  |
| 5. Pleased at 2016 election outcome | .01 | -.33^***^ | -.31^***^ | -.48^***^ | – |  |  |  |
| 6. Agreement with public recognition of legitimacy | .01 | -.30^***^ | -.12^*^ | -.28^***^ | .58^***^ | – |  |  |
| 7. News during | -.10^†^ | .001 | .09 | .08 | -.05 | -.02 | – |  |
| 8. News after | -.07 | .004 | .05 | .08 | -.03 | -.10 | .71^***^ | – |
| 9. Vote support (-1 = Clinton, 1 = Trump) | .01 | -.32^***^ | -.24^***^ | -.44^***^ | .87^***^ | .59^***^ | -.02 | -.03 |

## **Table S9. Descriptions of the Capitol storming by focus condition. (Study 5)**

| Republican Focus Condition | Fringe Focus Condition |
| --- | --- |
| Storming of the United States Capitol  The storming of the United States Capitol was a violent attack against the United States Congress at the US Capital. The goal of the crowd was to prevent the Electoral College vote count that would affirm Joe Biden as the winner of the recent presidential election.  Rioters assaulted Capitol police officers and reporters on the scene and attempted to locate, unsuccessfully, lawmakers who had legitimate reason to fear for their safety. Five people were fatally injured during the assault, including a police officer in the line of duty.  Although considerable media coverage blamed the attack on the anti-government militias and well-documented white supremacist groups that were involved, converging sources of evidence have largely shown that regular conservative citizens and others associated with the Republican party were heavily involved. Republican voters of every age were found to be present, along with some well-documented involvement of Republican party leaders including GOP Senator Doug Mastriano who promoted and attended the protest and Republican representative Derrick Evans who entered the Capitol with rioters. | Storming of the United States Capitol  The storming of the United States Capitol was a violent attack against the United States Congress at the US Capital. The goal of the crowd was to prevent the Electoral College vote count that would affirm Joe Biden as the winner of the recent presidential election.  Rioters assaulted Capitol police officers and reporters on the scene and attempted, unsuccessfully, to locate lawmakers who had legitimate reason to fear for their safety. Five people were fatally injured during the assault, including a police officer in the line of duty.  Although considerable media coverage blamed the attack on a few Republican leaders and regular conservative citizens, converging sources of evidence have largely shown that a number of extremist fringe groups unaffiliated with the Republican party were heavily involved. Anti-government militias such as the Oath Keepers and the Three Percenters were found to be present, along with some well-documented involvement of white supremacists such as Timothy Hale-Cusanelli and the costumed “QAnon shaman” Jacob Chansley. |

## **Table S10. Effects of political party and focus condition on responsibility for the Capitol storming.**

| Factor | *F, p, η_p_^2^* |
| --- | --- |
| **Extremist Fringe Groups** |  |
| Participant Political Party | *F*(1, 703) = 0.04, *p* = .852, *η_p_^2^* < 001 |
| Focus Condition | *F*(1, 703) = 2.16, *p* = .142, *η_p_^2^* = .003 |
| Participant Political Party × Focus Condition | *F*(1, 703) = 0.10, *p* = .757, *η_p_^2^* < 001 |
|  | |
| **Extremist Individuals** |  |
| Participant Political Party | *F*(1, 702) = 1.40, *p* = .238, *η_p_^2^* = .002 |
| Focus Condition | *F*(1, 702) = 0.40, *p* = .525, *η_p_^2^* = .001 |
| Participant Political Party × Focus Condition | *F*(1, 702) = 1.98, *p* = .160, *η_p_^2^* = .003 |
|  | |
| **Regular Republican Voters** |  |
| Participant Political Party | *F*(1, 702) = 370.07, *p* < .001, *η_p_^2^* = .345 |
| Focus Condition | *F*(1, 702) = 30.05, *p* < .001, *η_p_^2^* = .041 |
| Participant Political Party × Focus Condition | *F*(1, 702) = 0.17, *p* = .682, *η_p_^2^* < .001 |
|  |  |
| **Republican Political Leaders** |  |
| Participant Political Party | *F*(1, 702) = 900.41, *p* < .001, *η_p_^2^* = .562 |
| Focus Condition | *F*(1, 702) = 5.57, *p* = .019, *η_p_^2^* = .008 |
| Participant Political Party × Focus Condition | *F*(1, 702) = 5.03, *p* = .025, *η_p_^2^* = .345 |
| *Simple effect of focus condition on Republicans* | *F*(1, 702) = 9.92, *p* = .002, *η_p_^2^* = .014 |
| *Simple effect of focus condition on Democrats* | *F*(1, 702) = 0.01, *p* = .931, *η_p_^2^* < .001 |
|  | |
| **Regular Democrat Voters** |  |
| Participant Political Party | *F*(1, 702) = 128.34, *p* < .001, *η_p_^2^* = .155 |
| Focus Condition | *F*(1, 702) = 0.25, *p* = .617, *η_p_^2^* < .001 |
| Participant Political Party × Focus Condition | *F*(1, 702) = 0.62, *p* = .432, *η_p_^2^* = .001 |
|  | |
| **Democrat Political Leaders** |  |
| Participant Political Party | *F*(1, 702) = 383.38, *p* < .001, *η_p_^2^* = .353 |
| Focus Condition | *F*(1, 702) = 0.11, *p* =.742, *η_p_^2^* < .001 |
| Participant Political Party × Focus Condition | *F*(1, 702) = 0.79, *p* = .374, *η_p_^2^* = .001 |
|  | |
| **The Mainstream Media** |  |
| Participant Political Party | *F*(1, 701) = 281.55, *p* < .001, *η_p_^2^* = .287 |
| Focus Condition | *F*(1, 701) = 0.22, *p* = .640, *η_p_^2^* < .001 |
| Participant Political Party × Focus Condition | *F*(1, 701) = 1.38, *p* = .241, *η_p_^2^* = .002 |
|  | |
| **Social Media** |  |
| Participant Political Party | *F*(1, 702) = 12.00, *p* = .001, *η_p_^2^* = .017 |
| Focus Condition | *F*(1, 702) = 0.11, *p* = .742, *η_p_^2^* < .001 |
| Participant Political Party × Focus Condition | *F*(1, 702) = 2.05, *p* = .153, *η_p_^2^* = .003 |
|  | |
| **Misinformation** |  |
| Participant Political Party | *F*(1, 702) = 14.39, *p* < .001, *η_p_^2^* = .020 |
| Focus Condition | *F*(1, 702) = 0.01, *p* = .907, *η_p_^2^* < .001 |
| Participant Political Party × Focus Condition | *F*(1, 702) = 0.52, *p* = .472, *η_p_^2^* = .001 |

## **Table S11. Estimated marginal means and standard errors by cell for each of the measures of responsibility for the Capitol storming.**

|  | **Republicans** | | | **Democrats** | | | **Total** | |
| --- | --- | --- | --- | --- | --- | --- | --- | --- |
|  | **Repub. Focus** | **Fringe Focus** | **Total** | **Repub. Focus** | **Fringe**  **Focus** | **Total** | **Repub. Focus** | **Fringe**  **Focus** |
| **Extremist Fringe**  **Groups** | 7.91  (0.14) | 8.17  (0.16) | 8.04 (0.11) | 7.93  (0.14) | 8.10  (0.14) | 8.01  (0.10) | 7.92  (0.10) | 8.13  (0.10) |
| **Extremist Individuals** | 8.40  (0.13) | 8.67  (0.14) | 8.53  (0.10) | 8.42  (0.13) | 8.32  (0.13) | 8.37  (0.09) | 8.41  (0.09) | 8.50  (0.10) |
| **Regular Republican Voters** | 3.32  (0.17) | 2.30  (0.19) | 2.80  (0.13) | 6.57  (0.17) | 5.69  (0.16) | 6.13  (0.12) | 4.94  (0.12) | 3.99  (0.12) |
| **Republican**  **Leaders** | 4.01  (0.15) | 3.29  (0.17) | 3.65  (0.11) | 8.33  (0.15) | 8.31  (0.15) | 8.32  (0.11) | 6.12  (0.11) | 5.80  (0.11) |
| **Regular Democrat Voters** | 3.58  (0.16) | 3.37  (0.17) | 3.47  (0.12) | 1.66  (0.16) | 1.70  (0.15) | 1.68  (0.11) | 2.62  (0.11) | 2.54  (0.11) |
| **Democrat Leaders** | 5.35  (0.18) | 5.13  (0.19) | 5.24  (0.13) | 1.66  (0.18) | 1.76  (0.17) | 1.71  (0.12) | 3.51  (0.13) | 3.45  (0.13) |
| **The Mainstream**  **Media** | 7.06  (0.19) | 6.92  (0.20) | 6.99  (0.14) | 3.63  (0.19) | 3.94  (0.18) | 3.79  (0.13) | 5.34  (0.13) | 5.43  (0.14) |
| **Social**  **Media** | 7.34  (0.18) | 7.01  (0.20) | 7.18  (0.13) | 6.44  (0.18) | 6.64  (0.18) | 6.54  (0.13) | 6.89  (0.13) | 6.83  (0.13) |
| **Misinformation** | 7.30  (0.19) | 7.19  (0.20) | 7.25  (0.14) | 7.88  (0.19) | 8.04  (0.18) | 7.96  (0.13) | 7.59  (0.13) | 7.62  (0.14) |

Numbers in parentheses correspond to standard errors.

# **Supporting Figures**

## **Figure S1*.* Standardized Regression Coefficients for Moderated Mediation Analysis (Precursor Study 1)**

c and c’ represent the total and direct effects of the interaction between voter affiliation and candidate affiliation on current relevance.


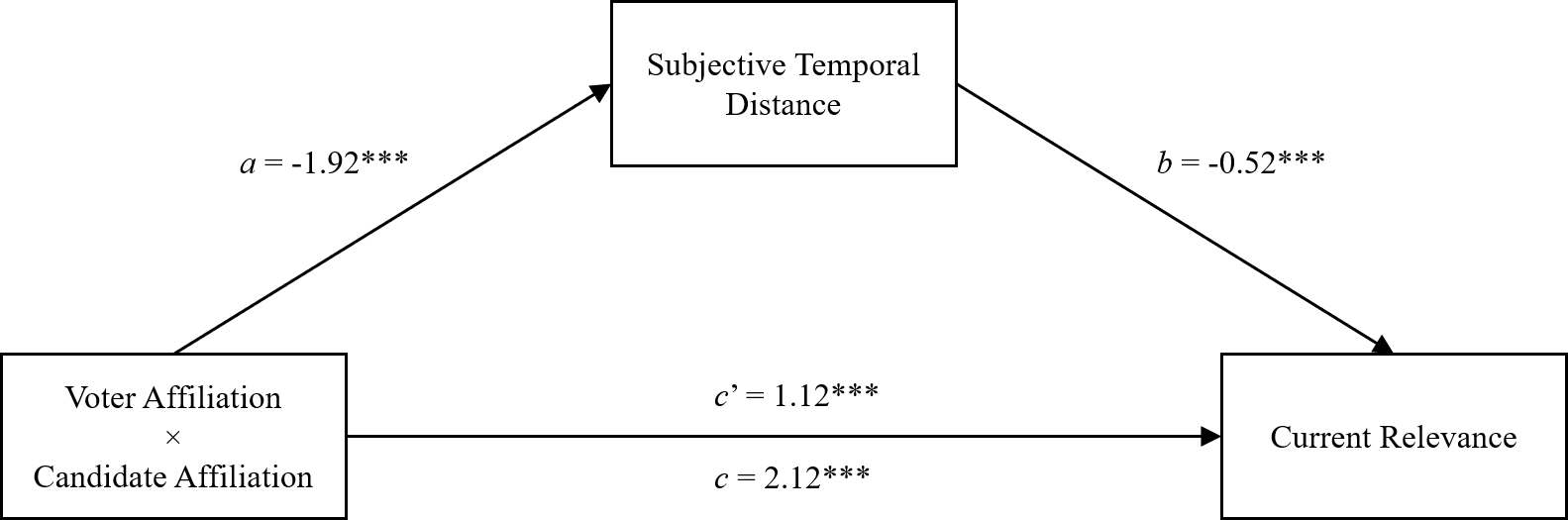


## **Figure S2*.* Standardized Regression Coefficients for Moderated Mediation Analysis (Precursor Study 2)**

c and c’ represent the total and direct effects of the interaction between voter affiliation and evaluation valence on current relevance.


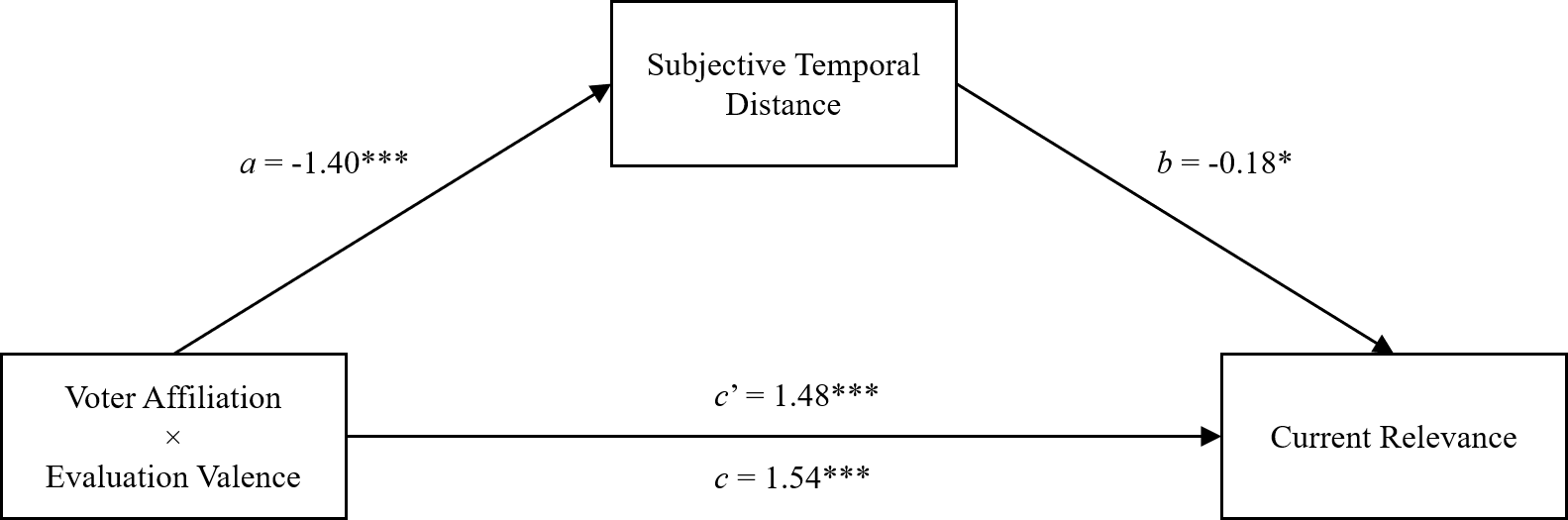


## **Figure S3*.* Standardized Regression Coefficients for Moderated Mediation Analysis (Precursor Study 3)**

c and c’ represent the total and direct effects of the interaction between voter group and evaluation valence on current relevance.


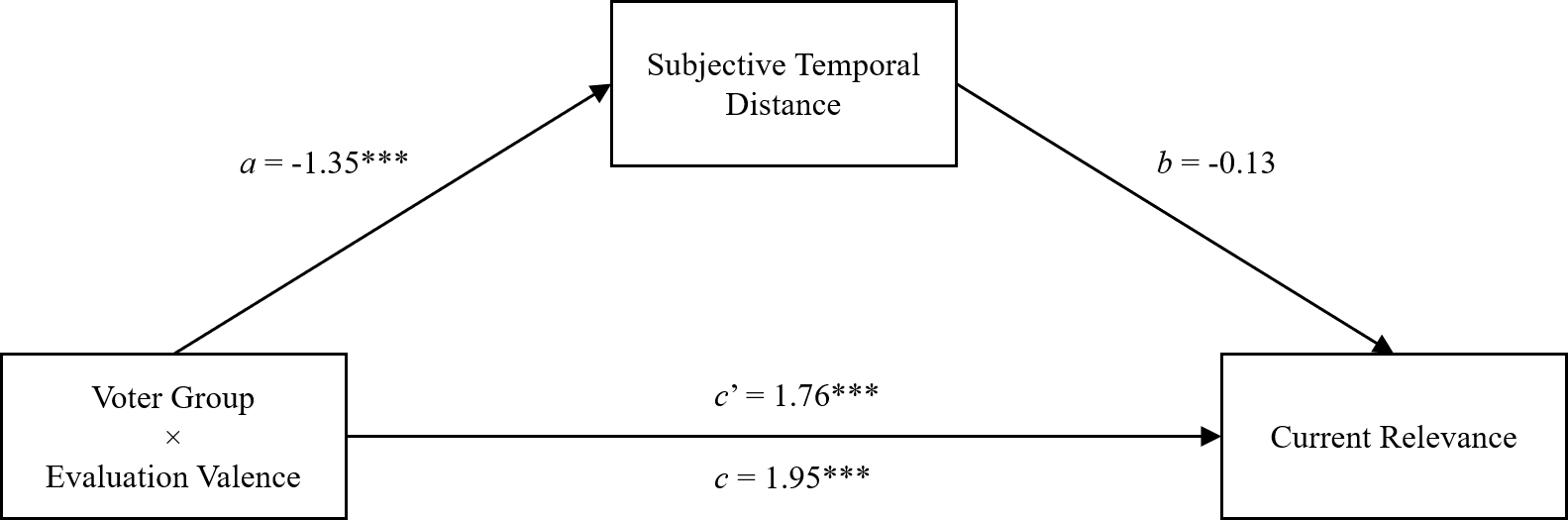


## **Fig S4. Standardized Regression Coefficients for Moderated Mediation Analysis*.***

c and c’ represent the total and direct effects of the interaction between voter affiliation, candidate affiliation, and event valence on current relevance.


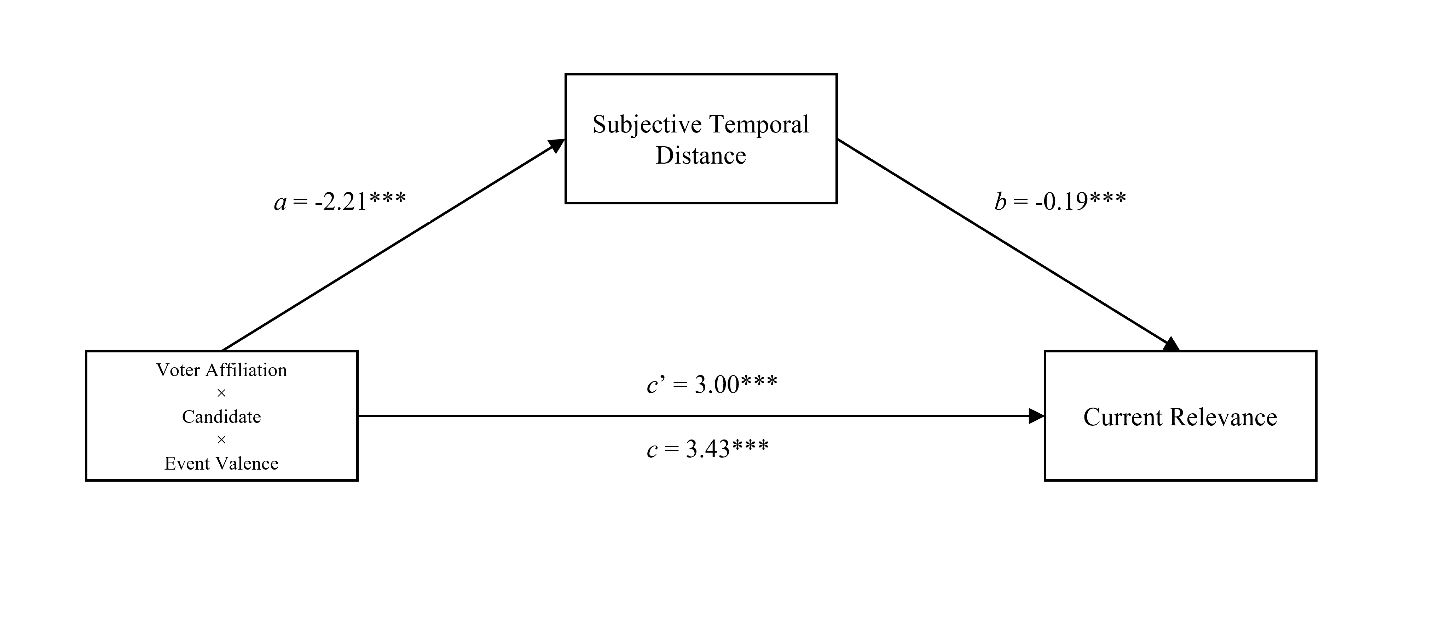


## **Fig S5. Standardized Regression Coefficients for Serial Mediation Analysis, Positive Review Condition.**

e and e’ represent the total and direct effects of voter group on voting intentions.


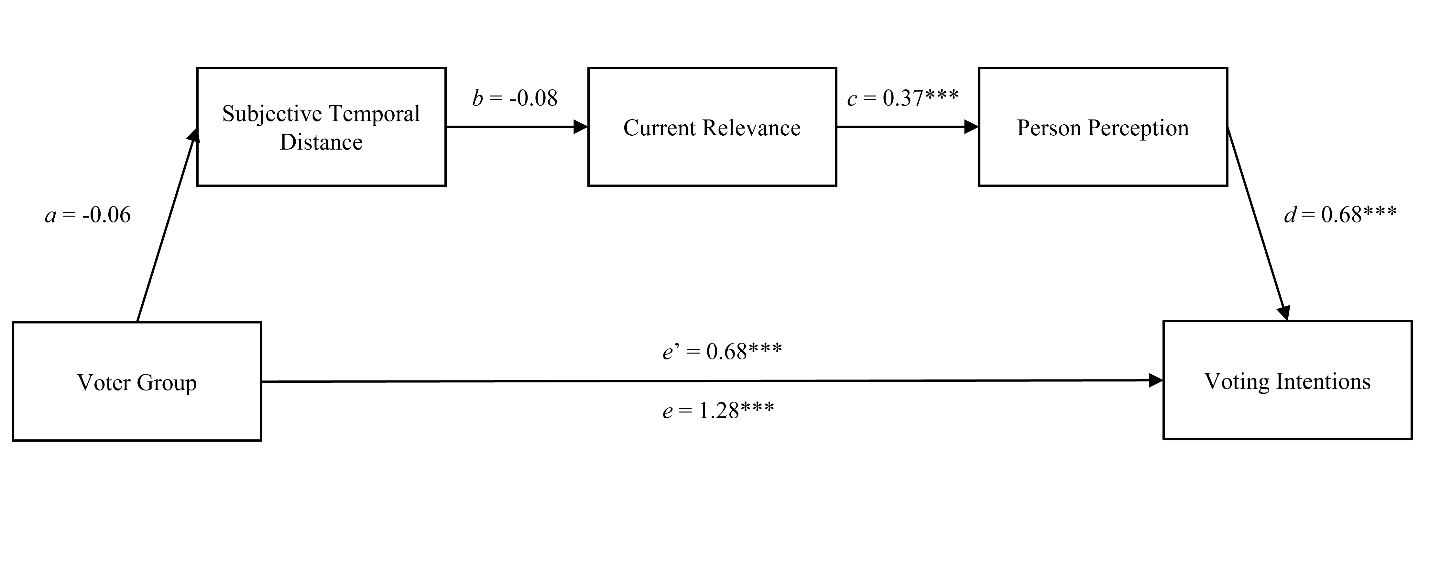


## **Fig S6. Standardized Regression Coefficients for Serial Mediation Analysis, Negative Review Condition.**

e and e’ represent the total and direct effects of voter group on voting intentions.


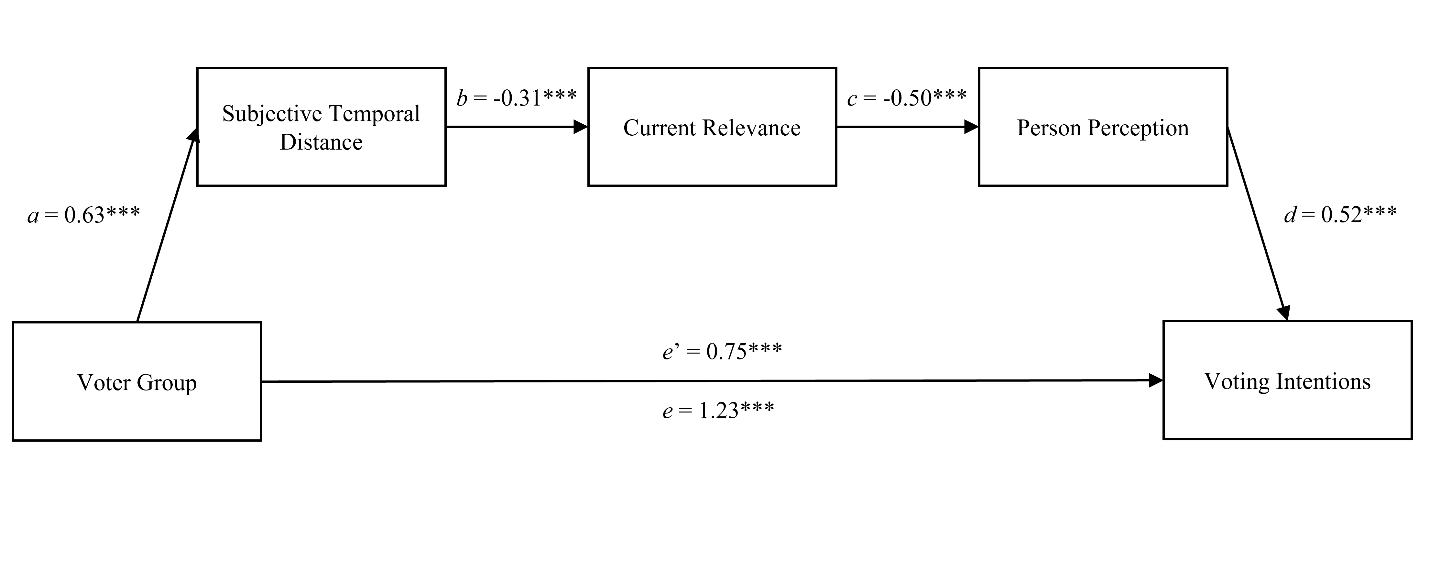


## **Fig S7. Standardized Regression Coefficients for Serial Mediation Analysis.**

e and e’ represent the total and direct effects of time condition on voting intentions.


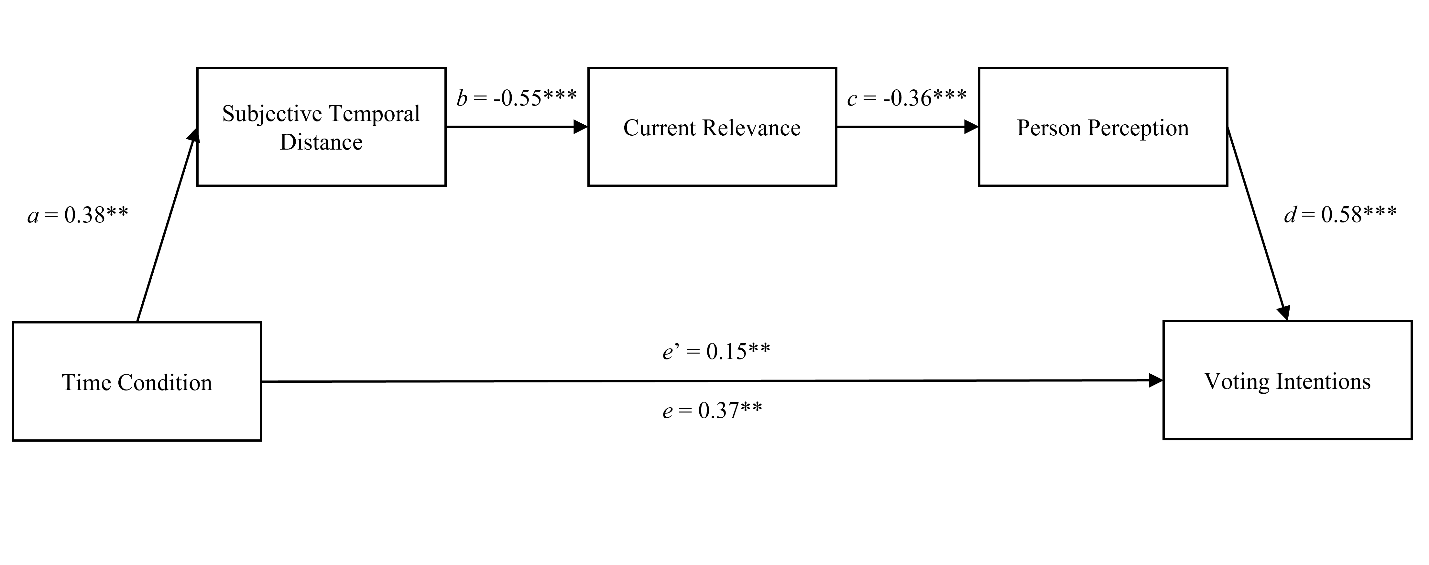


## **Fig S8. Standardized Regression Coefficients for Serial Mediation Analysis.**

d and d’ represent the total and direct effects of voter affiliation on Republican Party morality.


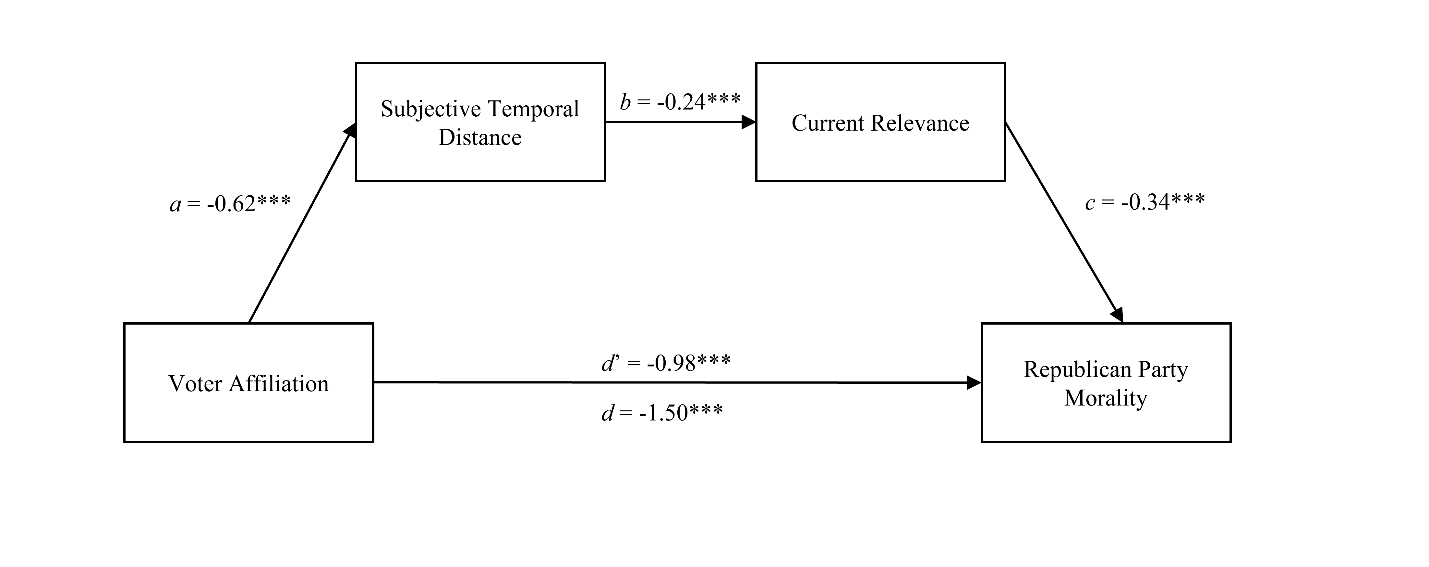


## **Fig S9. Relationship between affiliation strength/party pride and subjective temporal distance for Republicans and Democrats.**

Subjective temporal distance ranged from 0-100.


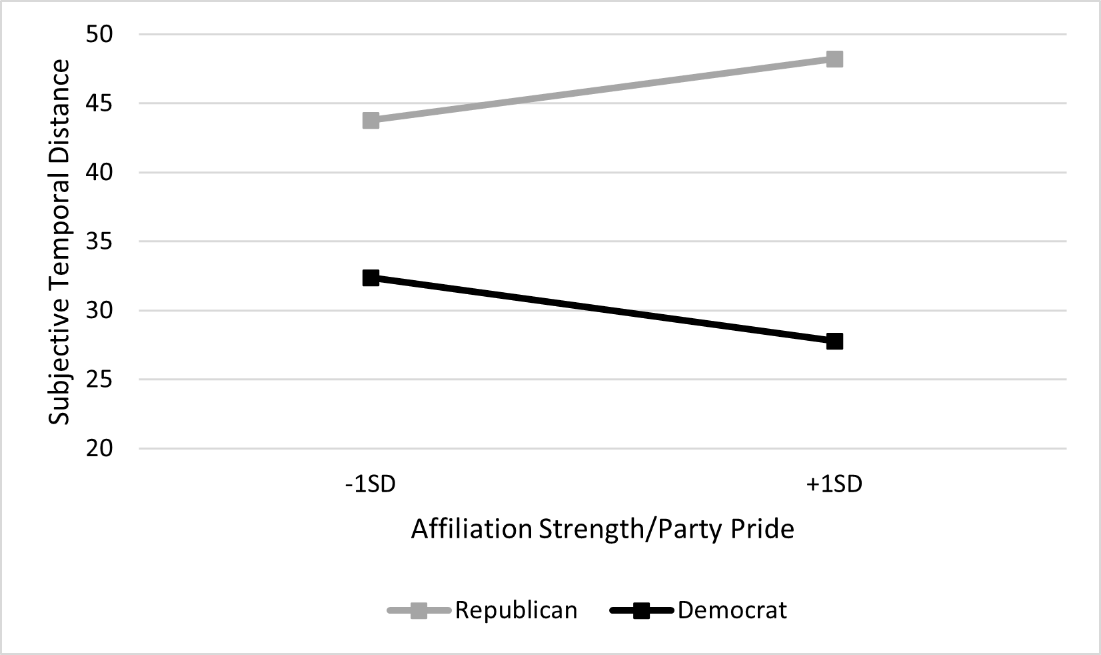


## **Figure S10. Relationship between affiliation strength/party pride and total current relevance for Republicans and Democrats.**


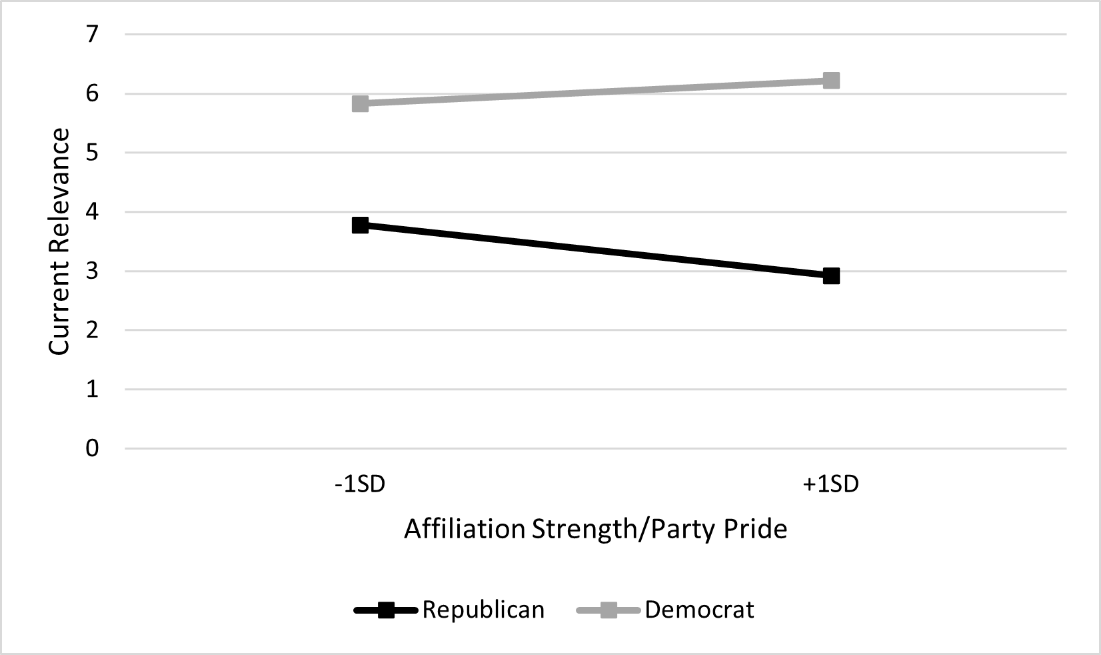

Supplement: S1 File — (DOCX) [file pone.0277179.s001.docx]
