# Supplementary material for: Far away or yesterday? Shifting perceptions of time for political ends
Source: PLoS One. 2022 Nov 3;17(11):e0277179. doi: 10.1371/journal.pone.0277179 (PMC9632895; doi:10.1371/journal.pone.0277179)
Supplement: Attachment — Submitted filename: Response to Reviewers October 2022.docx [file pone.0277179.s003.docx]

Dear Jeff Galak and the review team,

Thank you for your additional comments. Your feedback has further contributed to making sure our analysis and reporting is consistent and clear. In the letter below we include the text of your and reviewer’s feedback *in italics* followed by our responses immediately after each point.

Editor

*The three reviewers from the previous round read the paper again. Two of the reviewers recommended acceptance and one recommended a minor revision. I agree that there is still a small amount of work left, which is why I am considering this a minor revision. Please note that this means that I will NOT send the paper back out to review, but will rather make the final determination on my own. I encourage you to read over the reviews (especially R1) and address the final concerns that were raised. I don't anticipate that this will be a difficult hurdle for you.*

*Congratulations on an important contribution to science.*

We appreciate the work you have put in to ensure we received constructive feedback and to make the process clear and straightforward.

Reviewer #1

*I agree that subjective distance and subjective duration can be psychologically distinct, and that may explain the seemingly discrepant results in Study 2. That said, I don’t think removing the findings about subjective duration is an appropriate way to deal with the issue. I see it as a potentially failed prediction of the theory that can also be explained (post-hoc) by another mechanism, and I think it should be discussed as such, so that the reader can form their own interpretation.*

Thanks for flagging this. In re-reading this original section, on subjective duration and future temporal distance, we realized that we inadequately set up the exploratory nature of those measures and why we would not expect a parallel pattern, theoretically. We agree with your concerns that removing this section entirely would be the wrong approach, and that the reader should be presented the exploratory analysis allowing them to come to their own conclusions. However, we do explicitly highlight that we had no expectation that these measures would show the same pattern, given that the interaction was only produced by altering the perceived valence of a specific event (the election framed as flattering to Trump or questionable). We have no reason to think that this manipulation would affect voters’ ongoing experience of the Trump presidency (which Clinton voters would presumably find far more unpleasant than Trump voters), nor their predictions going forward to the next election. The original section did not do enough to make this reasoning clear. We have added more clarification, while still being careful to make it clear that any interpretation is wholly speculative. We have added the sections on subjective duration and distance from the 2020 election back into the manuscript (specifically pages 15-20 with tracked changes). On pages 15-16 and 18-19, we have also added short sections to clarify that these measures were exploratory and that we did not necessarily predict the exact same results. On page 19 we have added several sentences to explain why the results may have been different for these measures, focusing on the alternate mechanism by which aversive events feel slower in duration while pleasant experiences may “fly by.”

*Beyond the issue of response substitution, which I think has been reasonably addressed in this revision, there is a question of the interpretation of the effect. Specifically, it is unclear whether the observed findings are due to explicit motivated reasoning (e.g, temporal distancing as a tactic to minimize the impact of uncomfortable facts) or an implicit process outside of conscious awareness, or perhaps some form of dissonance reduction that might lie in between. I think the paper, as currently written, heavily implies an implicit process, without providing the evidence needed to draw that conclusion. First, I think it would be helpful to go through the paper and carefully consider the wording – the evidence only measures what people choose to report, not what they actually think or feel. Second, I think the speculation in the general discussion (“an innocent, subtle and possibly automatic process”, p. 35; “subtly shaped” and “potential subtle impact”, p. 36) is one-sided. I think it should be either be omitted or balanced out with a discussion of the opposite possibility (“a motivated, direct and possibly deliberate distortion”).*

You are correct that the previous draft suggested an implicit process without providing adequate reasoning. We have cut back on our use of this language, primarily from lines 779 to 796 with tracked changes in the general discussion. We have also added a short section towards the end of page 37 to make your point clear: “However, it is important to note that the current findings cannot conclusively determine the mechanism leading to these shifts in subjective time. For instance, we cannot determine with certainty whether the process is subtle and automatic or an explicit distortion – indeed, although people may not always be aware of how their experience of time shifts, they may well sometimes use distancing intentionally and strategically, as when political communicators publicly relegate missteps to the distant past. Determining more precisely the nature of the underlying processes behind shifting perceptions of time would be a beneficial avenue for future research.”

*I understand that Study 5 was pre-registered, but I was not able to find the pre-registration (I may have missed it). I didn't see a link in Study 5 or a posted pre-registration on OSF.*

It’s a good thing you pointed this out, because we had not given any clear directions to the preregistration in the previous draft. We have added a link to the OSF page in the introduction to Study 5 as well as in our data availability statement. Here is the link again for reference: <https://osf.io/3dvyc/?view_only=fe41bd0f690c40c782950749830047e7> The preregistration is not a built-in OSF registration but a word document in the OSF page named “IMPETuS Lab Archive Form Far Away or Yesterday Study 5 Final.docx”.

*Lastly, the OSF files include SAV and SPS files. I would recommend uploading .csv data files and a brief data dictionary explaining what each variable is and what the response codes are. I understand some of the survey materials are no longer available, but I think whatever materials can be found should be uploaded. Ideally, the OSF should contain all the information needed to either re-analyze the data or re-run the studies.*

In addition to the .sav and .sps files, we have included .csv files for each of the studies as well as data dictionaries on the OSF page. For those with access to SPSS, the easiest way to replicate our results would still be to run the syntax (sps) files that go through everything we report in the paper, but the new files should be sufficient for anyone who wants to recreate our analysis in another program.

As well, we have added pdf versions of the surveys for each of the main studies to the OSF page.

Reviewer 3

*The revised paper addressed almost all the point I raised. The only point that was ignored was that no table summarizing all the treatment was included. In the end, it is a matter of taste whether such a table is considered as useful, and the argument against the table is OK. The paper has gained in focus and the significant shortening was essential to improve the paper.*

We appreciate your understanding regarding the table. We hope the shortened paper will be effective in making things easy for readers to follow in a similar manner that you intended.

*I spotted a typo on line 791 (two periods) but apart from this I consider the paper as publishable.*

The typo has been fixed!

Thank you for your time and consideration,

Best Regards,

Andrew Dawson (on behalf of the research team)
